# A four-dimensional computational model of dynamic contrast-enhanced magnetic resonance imaging measurement of subtle blood-brain barrier leakage

**DOI:** 10.1016/j.neuroimage.2021.117786

**Published:** 2021-04-15

**Authors:** Jose Bernal, Maria d.C. Valdés-Hernández, Javier Escudero, Anna K. Heye, Eleni Sakka, Paul A. Armitage, Stephen Makin, Rhian M. Touyz, Joanna M. Wardlaw, Michael J. Thrippleton

**Affiliations:** a Centre for Clinical Brain Sciences, Dementia Research Institute, University of Edinburgh, Edinburgh EH16 4SB, UK; b School of Engineering, University of Edinburgh, Edinburgh EH9 3FB, UK; c Academic Unit of Radiology, University of Sheffield, Sheffield S10 2RX, UK; d University of Aberdeen, Centre for Rural Health, Inverness, UK; e Institute of Cardiovascular and Medical Sciences, University of Glasgow, Glasgow G12 8TA, UK

**Keywords:** Digital reference object, Blood-brain barrier permeability, DCE-MRI, Spatio-temporal imaging artefacts, Endothelial dysfunction, Cerebral small vessel disease

## Abstract

Dynamic contrast-enhanced MRI (DCE-MRI) is increasingly used to quantify and map the spatial distribution of blood-brain barrier (BBB) leakage in neurodegenerative disease, including cerebral small vessel disease and dementia. However, the subtle nature of leakage and resulting small signal changes make quantification challenging. While simplified one-dimensional simulations have probed the impact of noise, scanner drift, and model assumptions, the impact of spatio-temporal effects such as gross motion, *k*-space sampling and motion artefacts on parametric leakage maps has been overlooked. Moreover, evidence on which to base the design of imaging protocols is lacking due to practical difficulties and the lack of a reference method. To address these problems, we present an open-source computational model of the DCE-MRI acquisition process for generating four dimensional Digital Reference Objects (DROs), using a high-resolution brain atlas and incorporating realistic patient motion, extra-cerebral signals, noise and *k*-space sampling. Simulations using the DROs demonstrated a dominant influence of spatio-temporal effects on both the visual appearance of parameter maps and on measured tissue leakage rates. The computational model permits greater understanding of the sensitivity and limitations of subtle BBB leakage measurement and provides a non-invasive means of testing and optimising imaging protocols for future studies.

## Graphical abstract

**Figure:**
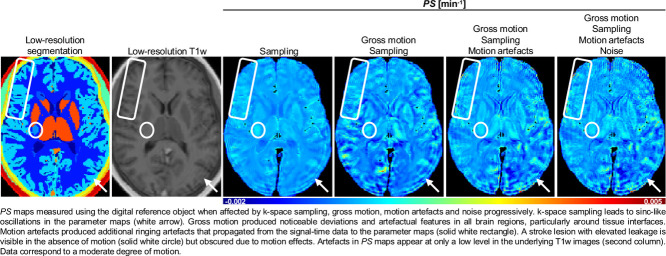

## 1 Introduction

DCE-MRI is the most commonly used technique for assessing breakdown of the blood-brain barrier (BBB) in neurological diseases, such as multiple sclerosis, brain tumours, stroke and small vessel diseases. By detecting the signal changes following intravenous injection of a gadolinium-based contrast agent (GBCA), quantitative estimates (*K*^Trans^ or *PS*) of its leakage across the BBB are obtained. While DCE-MRI is long-established in the context of high permeability, application of the technique is now rapidly growing in diseases such as cerebral small vessel diseases (SVD) and dementia, where BBB breakdown is typically very subtle. For example, recent studies have shown elevated BBB leakage in the normal-appearing white matter (NAWM) of patients with greater SVD burden, suggesting a possible role for BBB breakdown in the development of radiological signs and eventual clinical symptoms of the disease (Wardlaw et al., 2017). In the field of Alzheimer's disease, another recent study reported increased BBB leakage amongst APOE4 gene carriers, including those without cognitive impairment (Montagne et al., 2020).

Such advanced neuroimaging studies are highly valuable for understanding these diseases, whose pathophysiology is poorly understood (Wardlaw et al., 2019) and which have a major clinical and societal impact. However, while DCE-MRI is currently the standard imaging approach to investigating BBB dysfunction, the extremely low level of leakage and consequent small signal changes (typically a few percent) limit its accuracy and precision. Furthermore, the lack of a convenient reference method, and ethical and safety considerations around GBCA administration, make it difficult to assess measurement reliability and impede protocol optimisation, as summarised in two recent review and recommendation papers (Raja et al., 2018; Thrippleton et al., 2019). Previous computational studies have attempted to address this problem by applying a Monte-Carlo simulation approach to generate synthetic one-dimensional time-signal data for tissues with pre-specified leakage values, yielding important insights into the effects of pharmacokinetic model selection, signal stability and noise on the accuracy and precision of “permeability” mapping (Armitage et al., 2011; Barnes et al., 2016; Cramer and Larsson, 2014; Heye et al., 2016). However, this approach overlooks spatio-temporal effects, such as patient motion, partial volume effect and ringing artefacts, which may have a significant impact on the appearance of parameter maps (Heye et al., 2016) and, potentially, on reported leakage rates. Also, this method omits the contribution of extra-cerebral tissues, which typically exhibit a greater signal enhancement than brain tissues since they do not have a BBB, to imaging artefacts. The assessment of these spatio-temporal aspects is more conceptually and computationally demanding since both the measurement process and the participant must be simulated in the three spatial dimensions and time.

In this work, we propose an open-source computational model that uses *in-vivo* volunteer and patient data for mimicking the four-dimensional DCE-MRI acquisition process to evaluate the aforementioned confounds and enable better protocol optimisation in the future. We used a publically available high-resolution atlas to generate realistic head and neck anatomy (Iacono et al., 2015) and combined it with motion parameters and signal enhancement properties obtained from a large cohort of SVD patients (Wardlaw et al., 2017). We used the resulting digital reference objects (DROs) to simulate the appearance of leakage maps and measured leakage values in healthy and diseased brain tissue under realistic experimental conditions, including *k*-space sampling, noise, gross motion and motion artefacts. Finally, we explored the potential of image processing methods to enhance the accuracy of BBB leakage measurements.

## 2 Materials and methods

### 2.1 Digital reference object

We identified two main requirements for devising a realistic computational model for evaluating subtle BBB leakage measurement. First, it should contain both healthy and pathological brain tissues as well as non-brain tissues that are commonly excluded in simulations. Second, it should simulate aspects of the acquisition process that are known or expected to affect the appearance of DCE-MRI parameter maps, such as motion and truncation artefacts.

**Fig. 1:**
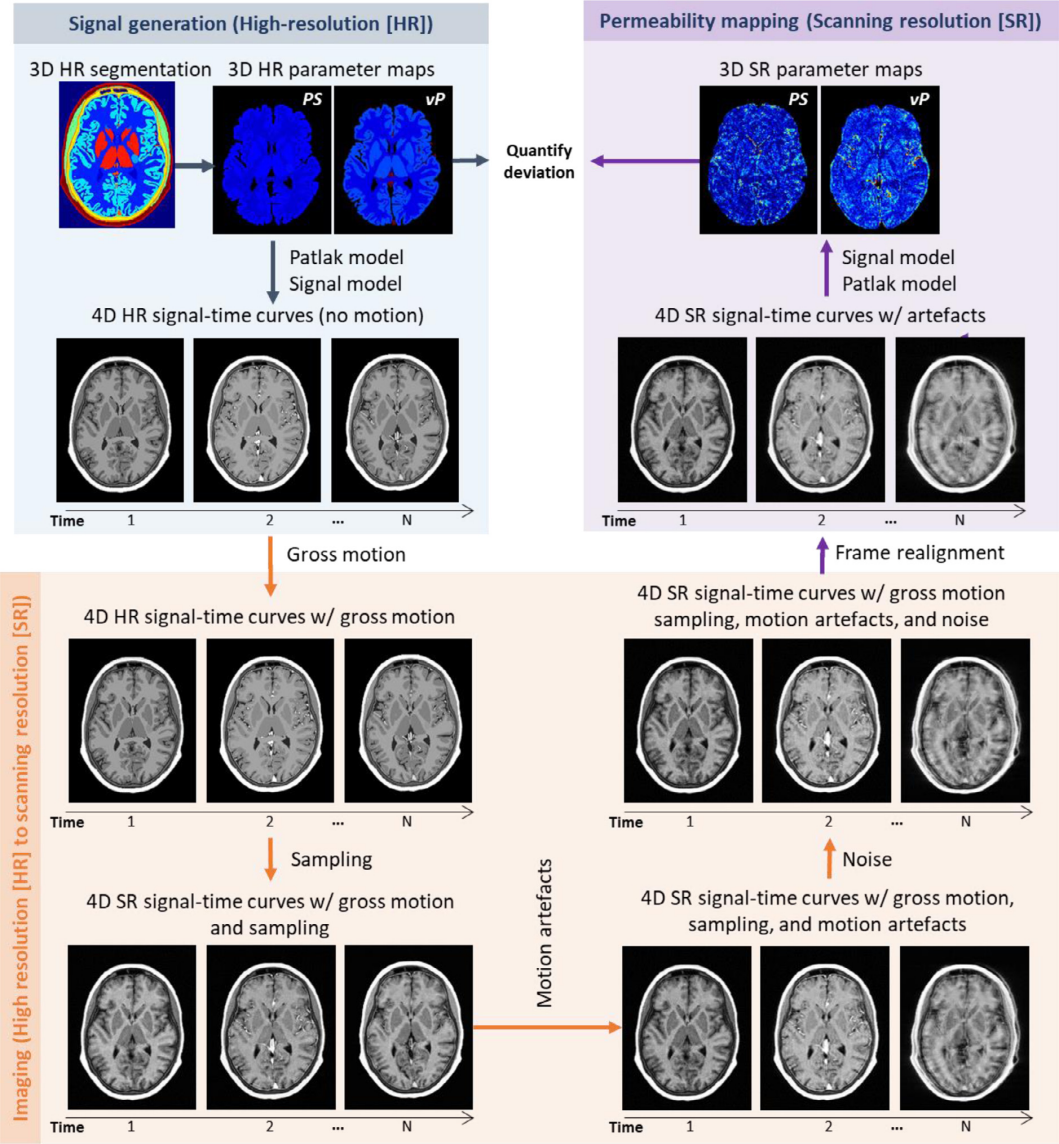
Proposed computational framework for generating 4D dynamic contrast-enhanced magnetic resonance images and simulating PS mapping under realistic conditions. PS: permeability-surface area product. v_P_: fractional blood plasma volume.

We simulated the DCE-MRI signal generation, measurement and analysis processes via the steps illustrated in Fig. 1 and described in detail below. Briefly, we used a high-resolution head model with pre-specified MR and ground-truth pharmacokinetic properties to generate four-dimensional time-signal data. We applied spatial transformations derived from *in-vivo* scan data to simulate gross motion and motion artefacts, resampled the resulting data in *k*-space and added random noise to yield “acquired” images at lower resolution. We post-processed and analysed these scans to obtain simulated pharmacokinetic parameter maps. Code for generating the DROs is freely available at https://doi.org/10.7488/ds/2966.

#### 2.1.1 Ground truth

**Fig. 2:**
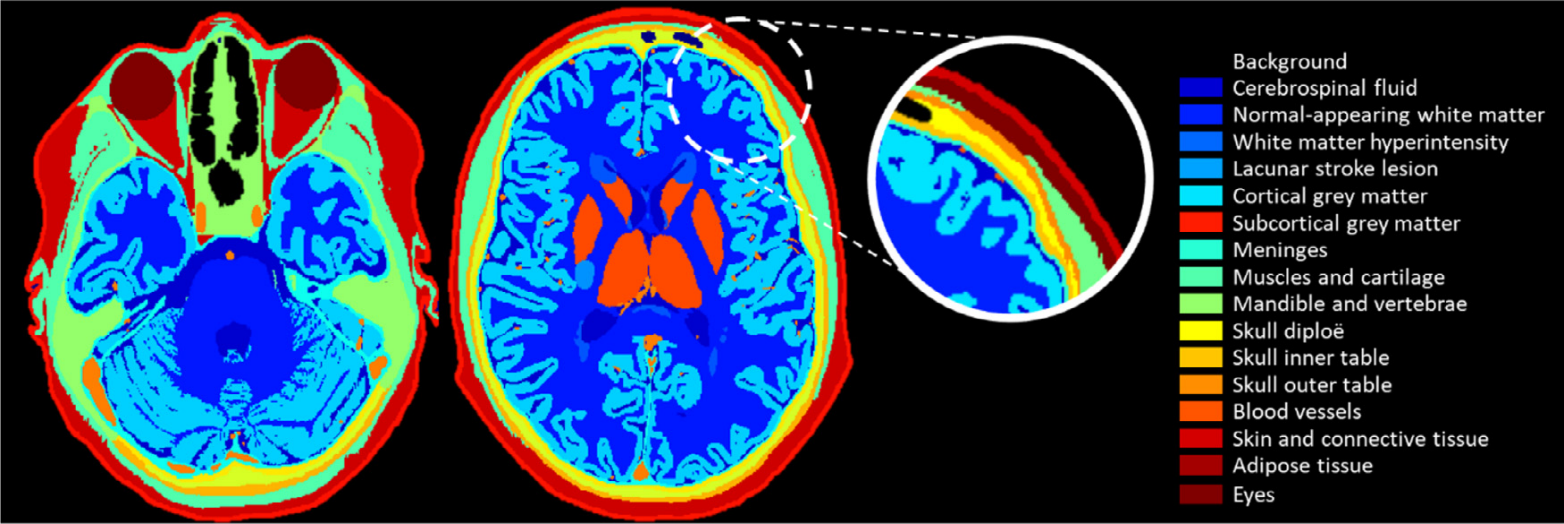
Tissue classes included in our computational model and used to generate 4D high-resolution digital reference objects. We considered 16 regions of interest, comprising cerebrospinal fluid; normal-appearing white matter; white matter hyperintensities; stroke lesion; cortical grey matter; subcortical grey matter; meninges; muscle and cartilage; mandible and vertebrae; skull diploe; skull inner table; skull outer table; blood vessels; skin and connective tissue; adipose tissue; and eyes. (For interpretation of the references to colour in this figure legend, the reader is referred to the web version of this article.)

We developed the signal model based on a three-dimensional high-resolution (0.5-mm isotropic), comprehensively-labelled and publicly-available human head and neck atlas (Iacono et al., 2015).^1^ The atlas is particularly suitable for application to DCE-MRI because a wide range of segmented tissues including both brain tissues and nearby GBCA-enhancing structures, such as vessels and muscle, are labelled (Fig. 2 and Figure S1). We combined some of the tissue classes to reduce complexity and because the enhancement properties of each are not well known. To better represent the ageing brain, we added two regions of neuropathology associated with elevated permeability, specifically white matter hyperintensities (WMH) and lacunar stroke lesions, using spatial occurrence templates extracted from patient data (https://doi.org/10.7488/ds/2716). The stroke lesion is based on that of a patient with one small recent lacunar stroke lesion in the basal ganglia. The total burden of WMH would be classified as Fazekas 2 periventricular WMH and Fazekas 1 deep WMH. In total, our computational model comprised 16 regions of interest. We assigned values for the equilibrium signal intensity *S*_0_, pre-contrast longitudinal relaxation time *T*_10_, blood plasma volume fraction *v*_P_ and permeability surface area product *PS* to each tissue class.

#### 2.1.2 Generation of high-resolution 4D signal

We simulated the GBCA concentration over time for brain voxels within the high-resolution reference object using the Patlak model (Patlak and Blasberg, 1985), which has previously been shown to closely describe tracer kinetic behaviour in the slow leakage regime at low temporal resolution (Heye et al., 2016; Larsson et al., 2009):

$$C_{t}\left[t\right]=v_{p}c_{p}\left[t\right]+PS\;\int_{0}^{t}c_{p}\left[t^{\prime }\right]\;dt^{\prime },$$

where *c*_p_[*t*] represents the specified GBCA time-concentration function in blood plasma or arterial input function (AIF) and *C*_t_[*t*] is the total tissue GBCA concentration. Although it would be possible to use a more complex pharmacokinetic model to simulate ground-truth concentration, by using the Patlak model we ensured that any errors in the fitted parameters are caused by the measurement process, which is the focus of this work. To simulate signal within tissues that do not have a BBB, such as muscle and skull, we used time-signal curves measured directly from in-vivo patient data, as described in the Supplementary Material.

Having computed the concentration-time curves per voxel, we calculated the corresponding signal-time curves using the spoiled gradient echo signal formula:

$$S\left[t\right]=S_{0}\frac{\left(1-e^{-TR/T_{1}\left[t\right]}\right)\sin\theta_{FA}}{1-e^{-TR/T_{1}\left[t\right]}\cos\theta_{FA}}\cdot e^{-TE/T_{2}^{*}\left[t\right]\;},$$

where TR and TE represent the repetition and echo times respectively, $\theta_{FA}$ is the excitation flip angle and $T_{2}^{*}\left[t\right]$ is the effective transverse relaxation time at time t. The relaxation rate was assumed to vary linearly with the contrast agent concentration:

$$\frac{1}{T_{i}\left[t\right]}=\frac{1}{T_{i0}}+r_{i}\cdot C_{t}\left[t\right],\;i=1,\;2^{*},$$

where *r*_i_ is the relaxivity. Time-signal curves were thus generated for each location within the three-dimensional high-resolution model.

#### 2.1.3 Simulation of acquired data and motion effects

We considered the following steps to simulate DCE-MRI data acquisition:

*Starting head position*: First, we randomised the initial head position by applying a rigid-body spatial transform to the high-resolution DRO, such that each degree of freedom was randomly distributed with uniform probability over the range ±5° for rotations and ±2.5 mm for translations. This initial transformation was the same for all time frames.

*Gross patient movement*: Second, we simulated gross patient movement during the subsequent DCE-MRI acquisition by applying a different rigid-body transformation to the high-resolution DRO at each time frame. Movement trajectories for each of 201 simulation runs were based on in-vivo patient imaging data as described in Section 2.2.1.

*k-space sampling*: Third, we calculated the *k*-space representation of the high-resolution DRO for each time frame as the three-dimensional inverse Fourier transform. We resampled it to obtain *k*-space data with the acquired field of view and spatial resolution. We assumed three-dimensional Cartesian *k*-space sampling and suppressed signals from outside the field of view in the frequency- and slice-encoding directions to simulate band filtering and slab-selective excitation, respectively. The finite sampling of *k*-space results in information loss, which may manifest as Gibbs ringing artefacts and partial volume effects.

*Motion artefacts*: Fourth, we simulated motion artefacts using the approach of Shaw et al. (2020). In a nutshell, we generated a composite *k*-space image for each frame in which a random proportion of the successive *k*-space lines were acquired with the head in its initial position (i.e. that at the end of the previous time frame) and the remaining lines acquired with the head in its subsequent position (i.e. that at the start of the next time frame). The level of displacement between consecutive frames and the time at which the motion occurs determines the severity and appearance of the motion artefacts in the resulting image, typically manifesting as blurring, ringing and/or ghosting.

*Noise and fourier transformation*: Fifth, we added uncorrelated additive white Gaussian noise to the real and imaginary channels of the sampled *k*-space image, applied a three-dimensional Fourier transform and computed the magnitude to yield the “acquired” four-dimensional DRO image including sampling artefacts, gross motion, motion artefacts and Rician noise.

#### 2.1.4 Analysis of simulated DRO images

We processed the simulated DCE-MRI data following the approach described previously in (Heye et al., 2016). Briefly, we spatially realigned all frames using MCFLIRT (Jenkinson et al., 2002), computed enhancement and GBCA concentration profiles for each voxel, and fitted time-concentration curves to the Patlak model using multiple-linear regression to obtain voxelwise maps of estimated *v*_P_ and *PS*. We have calculated all results by omitting the first three post-contrast time points from the model fitting, to replicate the in-vivo data analysis process, as implemented in our previous study.

To obtain summary parametric measures for each tissue, we performed the following steps to generate the segmentation map in the acquired image space. First, we registered the high-resolution pre-contrast T1w image to the acquired one. Second, we applied the resulting affine transformation matrix to the binary mask of each tissue class, interpolating with a cubic approximation. Third, for each voxel, we assigned the label corresponding to the tissue class with the maximum probability, resulting in a region of interest (ROI) binary mask for each tissue.

Using the produced segmentation map, we generated a *T*_10_ map at the acquired resolution using the average region-wise values extracted from our patient cohort. For each tissue we calculated mean and median *v*_P_ and *PS* values using two approaches: fitting of the ROI-averaged signal and averaging over the parametric maps.

### 2.2 In-silico experiments

#### 2.2.1 MR protocol parameters and input data for generating DROs

We explored the qualitative impact of experimental factors on leakage mapping and the quantitative impact on parameter estimates using DCE-MRI data simulated using the framework described above. As a representative MR protocol used for measuring low-level BBB leakage, we simulated the acquisition protocol used in a recent DCE-MRI study of SVD patients (Wardlaw et al., 2017). Briefly, 201 patients with lacunar or cortical mild stroke recruited prospectively in the Mild Stroke Study 2 (MSS2) were scanned at 1.5 T using a 3D T1-w spoiled gradient echo sequence (21 time points, intravenous bolus injection of 0.1 mmol/kg gadoterate meglumine) for DCE-MRI, in addition to *T*_10_ measurement. Specific information about the reference imaging protocol is condensed in Table 1. The patients had a wide range of extents of neuroimaging features of SVD and were carefully phenotyped at presentation, recruitment and at up to three years of follow-up. The in-vivo study was conducted following Research Ethics Committee approval (ref. 09/81,101/54) and according to the principles expressed in the Declaration of Helsinki; all patients gave written informed consent. Full details of the study protocol, image acquisition and processing, and results are given in (Heye et al., 2016; Valdés Hernández et al., 2015; Wardlaw et al., 2017).

**Table 1:** MR protocol parameters used in our simulations. The values of these parameters are the same as those used in the Mild Stroke Study 2 (Heye et al., 2016).

MR protocol parameter	Parameter value
Repetition time	8.24 ms
Echo time	3.1 ms
Flip angle	12°
Field of view	24 × 24 × 18.4 cm
Acquired resolution	0.9375 × 1.25 × 4 mm
Temporal resolution	73 s
Pre-contrast acquisitions	1
Post-contrast acquisitions	20

We used average *S*_0_, *T*_10_, *v*_P_ and *PS* values for NAWM, grey matter, recent stroke lesion and WMH obtained from this study as ground-truth values for generation of DRO signals. The values used in the simulations can be found in Table 2. For vessels, we used *PS* = 0 and *v*_P_ = 1-Hct (Hct was assumed to be 0.45). Given that the Patlak model does not reflect the enhancement over time of extra-cerebral regions, we sampled their signal-time curves from in-vivo images manually, under the supervision of an experienced neuroradiologist, and fitted the resulting curves using exponential or power functions to remove noise. The fitted functions can be found in the Supplementary Material. We extracted the signal intensity profiles of meninges, skin and muscle, mandible and vertebrae, eyes, and skull diploe, inner and outer tables from DCE-MRI scans of a single patient in the study cohort. We used a population-average vascular input function derived from the same data (Heye et al., 2016).

**Table 2:** Tissue parameters used as ground truth values in our simulations. Equilibrium signal, *S*_0_, pre-contrast longitudinal relaxation time, *T*_10_, blood-brain barrier permeability-surface area product, *PS*, and capillary blood plasma volume fraction, *v*_P_ are median values measured in the Mild Stroke Study 2 (Heye, 2015). To simulate a stroke lesion with high leakage rate, we used the third quartile of the *PS* and *v*_P_ distribution measured in the same study.

Tissue class	*S*_0_	*T*_10_ (s)	*PS* (×10^−4^min^−1^)	*v*_P_ (×10^−2^)
Normal appearing-white matter	9726	0.99	2.75	0.57
White matter hyperintensity	9402	1.20	3.91	0.72
Grey matter	9298	1.34	3.85	1.20
Recent stroke lesion	9858	1.27	7.25	1.05

We used distinct motion trajectories for each run to generate realistic motion effects in our simulations, including examples of low, moderate and high degrees of motion. The classification of motion trajectories based on the MSS2 data can be found in the Supplementary Material. A trajectory consisted of 20 rigid-body transformation matrices, where each matrix is the inverse of the transformation used to realign each time frame in the MSS2. We generated 201 DROs using a different motion trajectory, randomised starting position and newly-generated spatial noise for each run.

To achieve a realistic spatial noise level, we measured an in-vivo spatial signal-to-noise ratio of 91.5 for NAWM and applied the corresponding noise level (scaled to match the simulated pre-contrast NAWM signal) to all voxels in the simulated images.

#### 2.2.2 Experiments and reporting

We generated and analysed synthetic DCE-MRI data for a representative SVD patient as described above. We first performed *in-silico* experiments to investigate the impact of *k*-space sampling, gross motion, motion artefacts and noise on the appearance of parametric maps. To further investigate these effects, we performed simulations for different initial head positions and degrees of head motion; to investigate the influence of non-brain GBCA uptake, we ran additional experiments with and without enhancement of extra-cerebral tissues. To evaluate the possible impact on study findings, we generated and analysed 201 DROs, determining measured *PS* values for each DRO and tissue, using multiple processing approaches (statistics: mean signal, median signal, mean parameter, median parameter; post-processing: segmentation mask erosion, spatial realignment, low-pass spatial filtering). For each approach, we obtained the *PS* distribution across all 201 DROs, reporting the median and interquartile range (IQR) values (RStudio v1.2.5019 with R v3.5.1).

We ran all experiments on a 189GB RAM computer running Scientific Linux 7.3 (Nitrogen; Arch x86 64; 56 CPUs Intel(R) Xeon(R) CPU E5-2683 v3 @ 2.00 GHz). Each simulation took approximately 30 min.

## 3 Results

### 3.1 Qualitative appearance of parameter maps

**Fig. 3:**
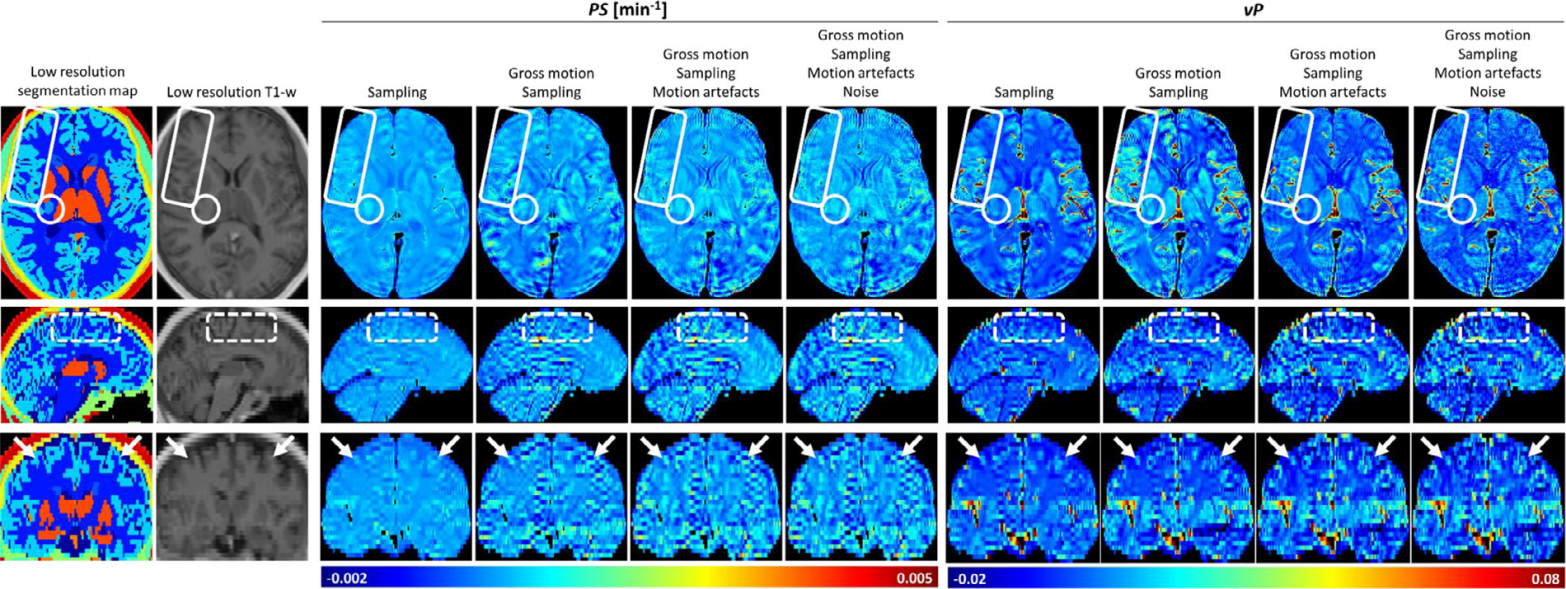
*PS* and *v*_P_ maps measured using the DRO when affected by *k*-space sampling, gross motion, motion artefacts and noise progressively. *k*-space sampling leads to sinc-like oscillations in the parameter maps (white arrows in coronal view), which are particularly evident in the z-direction (superior to inferior). Gross motion produced noticeable deviations and artefactual features in all brain regions, particularly around tissue interfaces (white dashed rectangle in sagittal view). Motion artefacts produced additional ringing artefacts that propagated from the signal-time data to the *PS* maps (solid white rectangle in axial view). A stroke lesion with elevated leakage is visible in the absence of motion (solid white circle in axial view) but obscured due to motion effects. Note that the artefacts observed in the *PS* maps appear at only a low level in the underlying T1w images (second column). Data correspond to a moderate degree of motion. (For interpretation of the references to colour in this figure legend, the reader is referred to the web version of this article.)

**Fig. 4:**
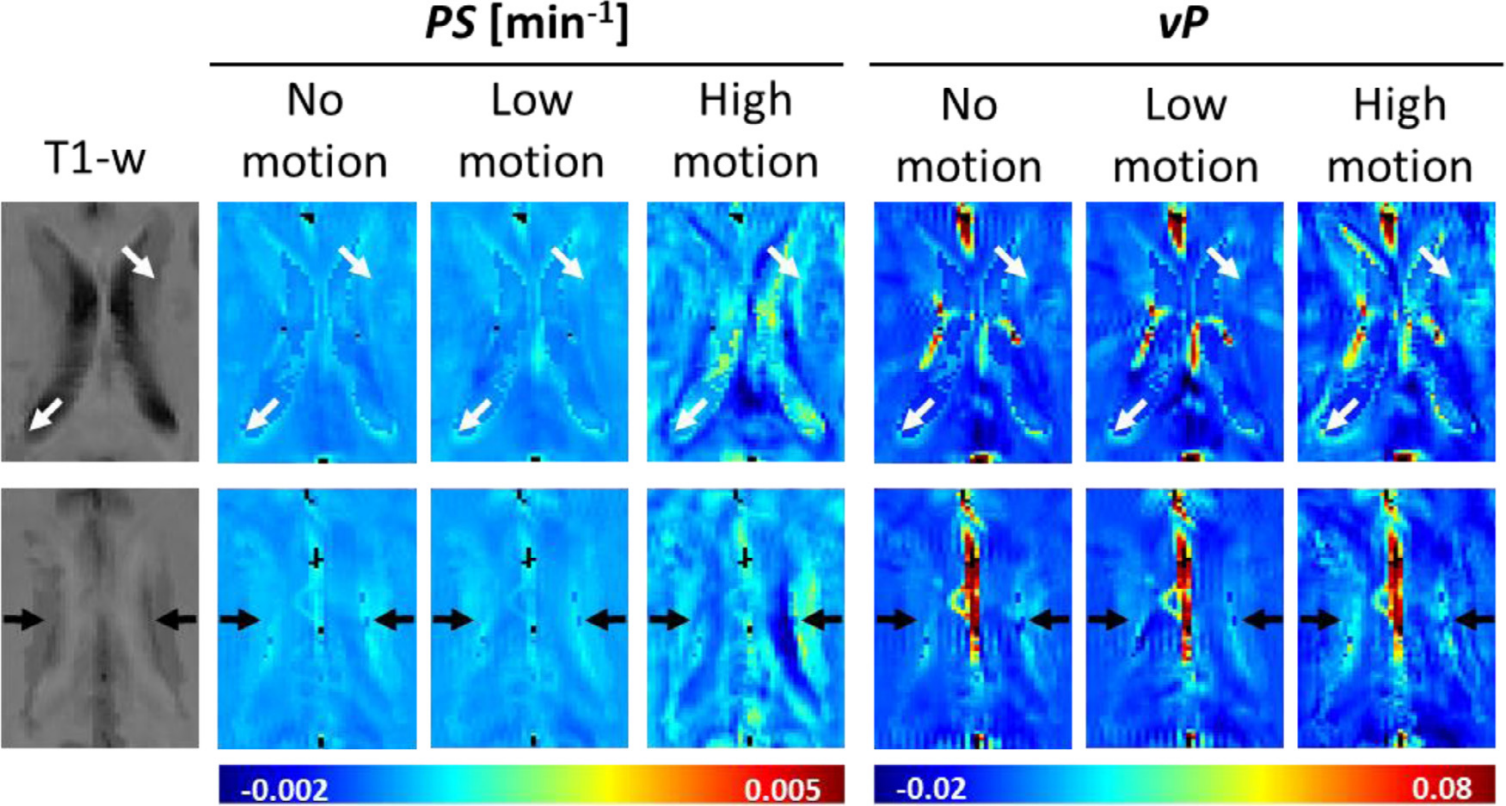
Artefactual periventricular leakage due to sampling and motion effects. The artefactual features are evident around the lateral ventricles (white arrows) and in the adjacent supraventricular corona radiata (black arrows), where white matter hyperintensities frequently occur. Such artefacts, caused by sampling and motion effects, might potentially be confused with “periventricular BBB dysfunction” due to subtle pathology.

Fig. 3 shows parametric maps measured from a generated DRO and showing the cumulative effects of sampling (i.e. truncation in *k*-space), gross patient motion, motion artefact and noise. The DRO was generated assuming uniform *PS* and *v_P_* within each brain tissue class. Data sampling results in ringing artefacts in all three spatial dimensions, particularly in the slice direction where the voxel dimension is highest; nevertheless, differential leakage between tissues is resolved, including elevated *PS* in the WMHs and stroke lesion and elevated *v_P_* in the grey matter and stroke lesion. However, inclusion of moderate patient motion obscures tissue differences and induces artefactual features close to tissue boundaries and vessels, and additional ringing artefacts. The additional effect of noise is small, with motion effects dominating the visual appearance. It is noteworthy that, while the motion effects on parameter maps are severe, the degree of head motion is moderate and the impact of artefacts on the underlying T1w images is seen to be small in comparison to that on the parameter maps. To further illustrate the implications of motion, Fig. 4 shows simulated *PS* and *v_P_* maps in the basal ganglia region, where areas of falsely increased (and decreased) periventricular leakage appear and become more apparent with increasing degree of head movement.

**Fig. 5:**
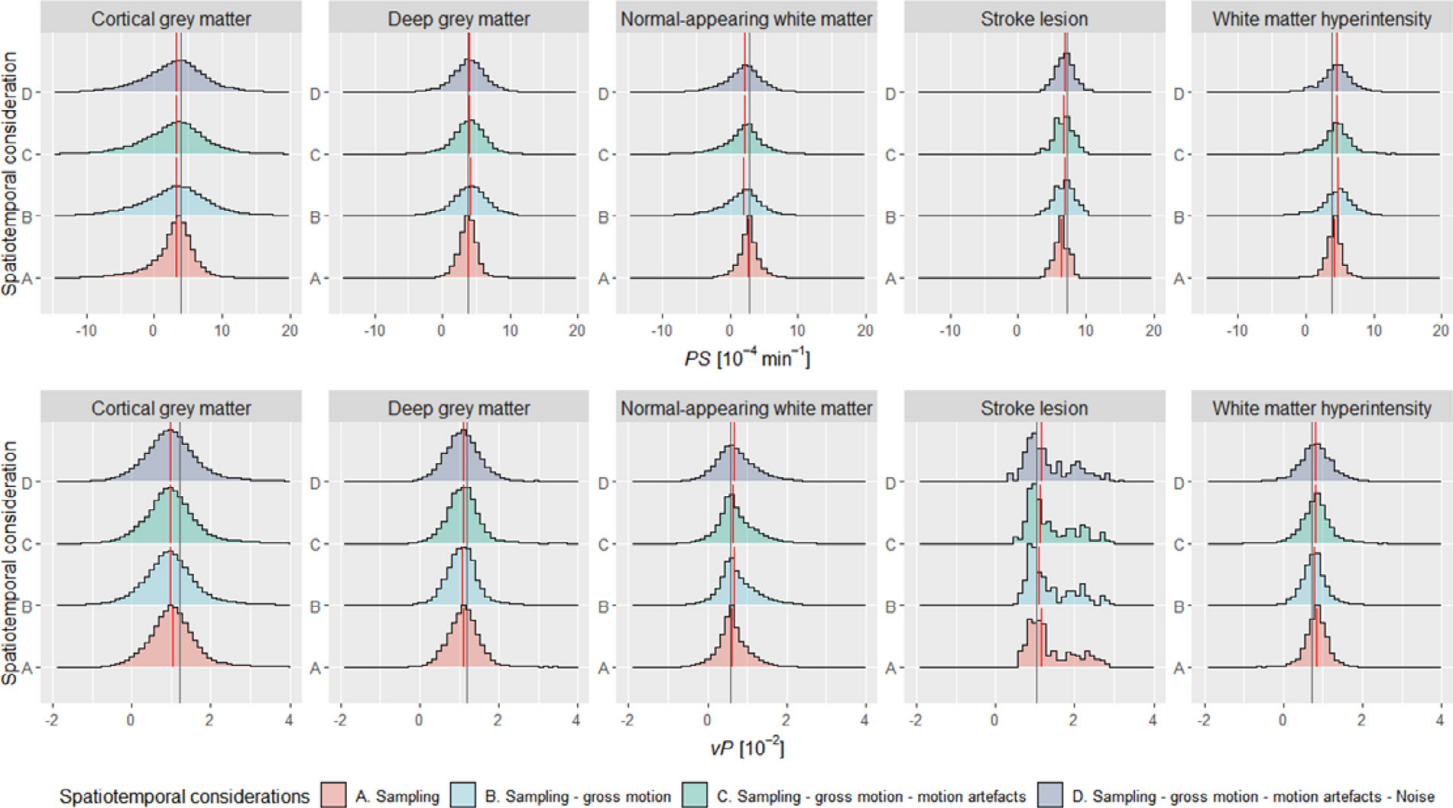
Distribution of measured *PS* and *v*_P_ values for each tissue of interest as we induced spatio-temporal effects progressively in a single patient (data correspond to ‘Low motion’ images shown in Fig. 4). The vertical lines depict the ground truth (grey) and median (red) parameter values. We used the term “sampling” to refer to *k*-space sampling, which results in information loss and manifests as partial volume effects and Gibbs ringing artefacts. (For interpretation of the references to colour in this figure legend, the reader is referred to the web version of this article.)

To quantify these effects, we created histograms to show the distribution of *PS* and *v*_P_ within each tissue for a single DRO (simulated with low-level patient motion, Fig. 5). Although we defined each tissue to have a uniform *PS* and *v_P_, k*-space sampling generates a broad distribution of values, which becomes broader when we incorporate motion and noise in the DRO. In addition to causing a distribution of values, both sampling and gross motion affect central tendency values (i.e. mean or median), especially for cortical grey matter where the bias in *PS* and *v_P_* values was approximately −0.71 × 10^−4^ min^−1^ and −0.20 × 10^−2^ (–18.49% and −16.66%) relative to the ground-truth value, respectively. Spatiotemporal imaging considerations affect *PS* mapping more than *v_P_* mapping. The effects simulated can also result in non-physical negative *PS* and *v_P_* values in some voxels.

### 3.2 Effect of head position and extra-cerebral tissue enhancement

**Fig. 6:**
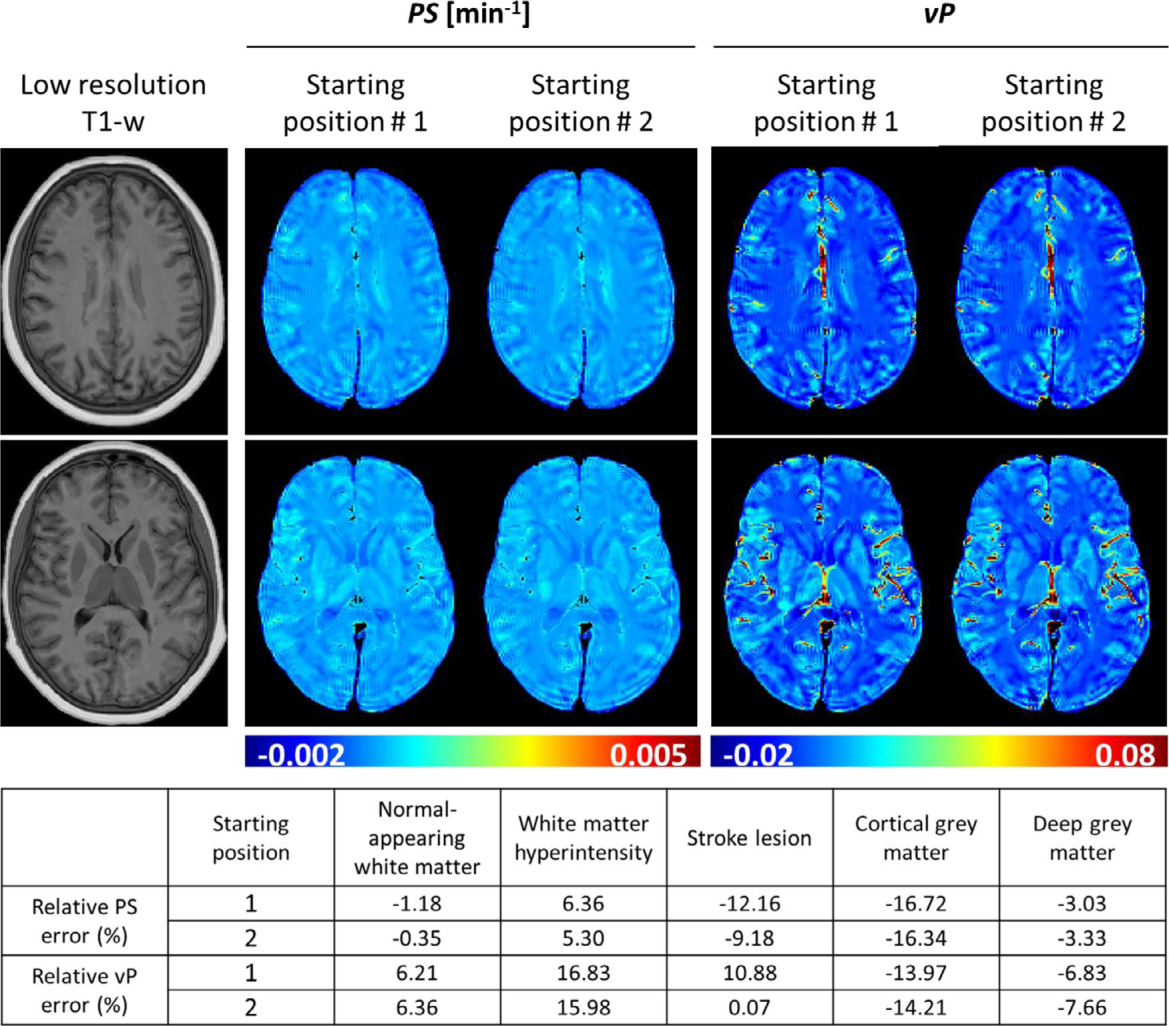
Gibbs-like artefacts in the *PS* and *v*_P_ parameter maps depend on initial head position, consistent with *k*-space sampling effects. Simulations excluded motion and noise effects.

The spatial relationship between the voxel grid and the head position determines the appearance of partial volumes and Gibbs ringing artefacts (Kellner et al., 2016). We simulated the impact of this effect on leakage mapping by analysing the error after randomising the starting head position (without intra- or inter-frame motion or noise). Qualitative results of this experiment for two DROs are shown in Fig. 6. The error caused by sampling the signal in *k*-space propagates differently depending on the initial head position, causing both over- and under-estimation of the actual parameter values in for the two initial positions.

**Fig. 7:**
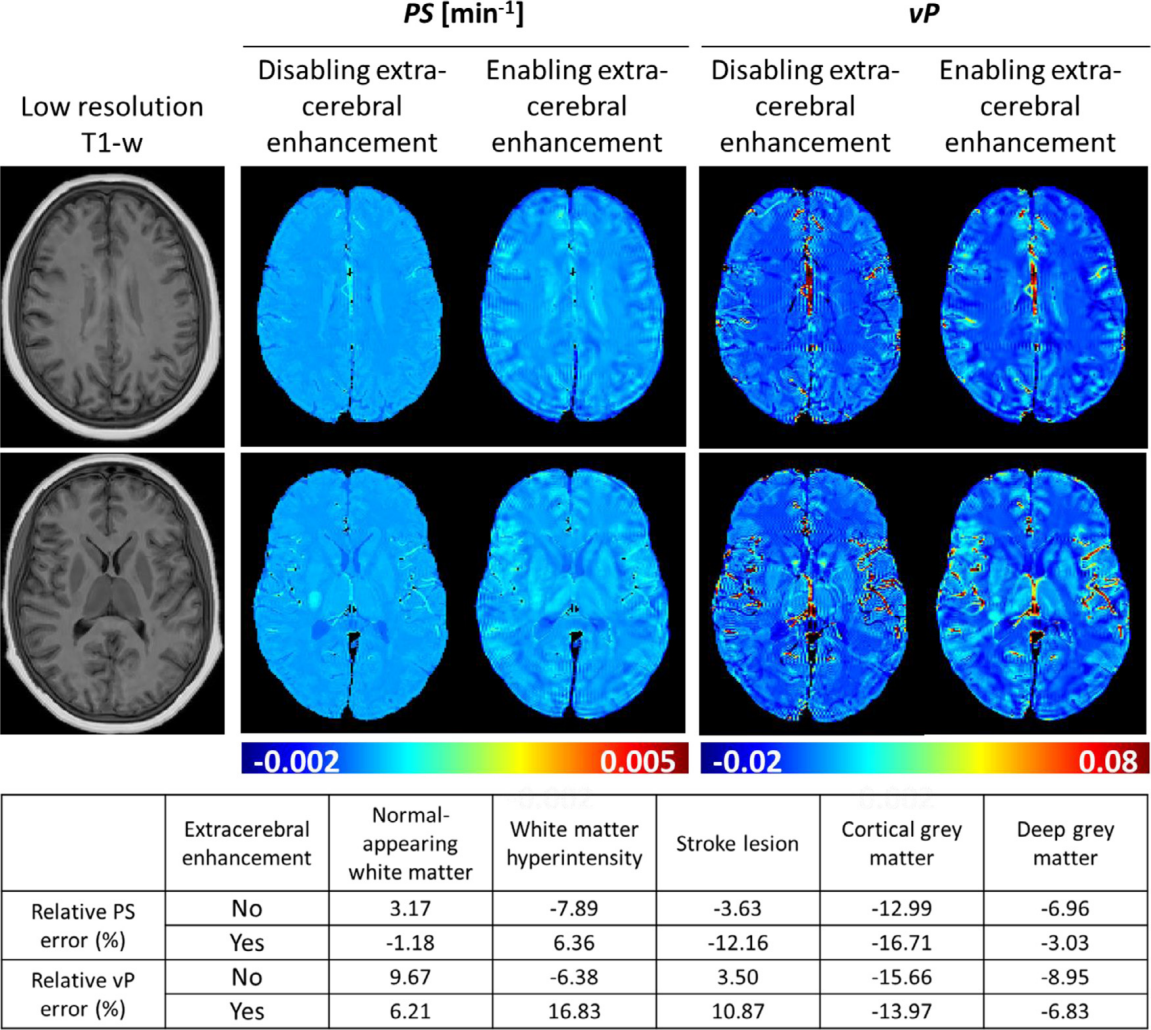
Effect of *k*-space sampling and extra-cerebral tissue enhancement on parameter mapping. The sinc-like artefacts in the parameter maps disappear when non-brain tissue enhancement is disabled, suggesting that such errors are caused by sampling of the high spatial frequencies induced by extra-cerebral enhancement. Simulations excluded motion and noise effects.

To address the impact of extra-cerebral tissue enhancement on *PS* and *v*_P_ parameter maps, we performed simulations with and without non-brain signal enhancement. The qualitative results for a single DRO shown in Fig. 7 demonstrate that extra-cerebral signal enhancement is responsible for the observed sinc-like artefacts as they attenuated when we disabled extra-cerebral enhancement. Residual errors persisted in tissue proximal to medium and large blood vessels. These findings were reproducible across a range of head starting positions. Quantitative tissue parameter estimates for the individual scan simulated in Fig. 7 were affected by up to 17% (note that the errors shown arise from multiple sources and are not necessarily reduced when extra-cerebral enhancement is disabled).

### 3.3 Accuracy and precision of quantitative parameter estimation

**Fig. 8:**
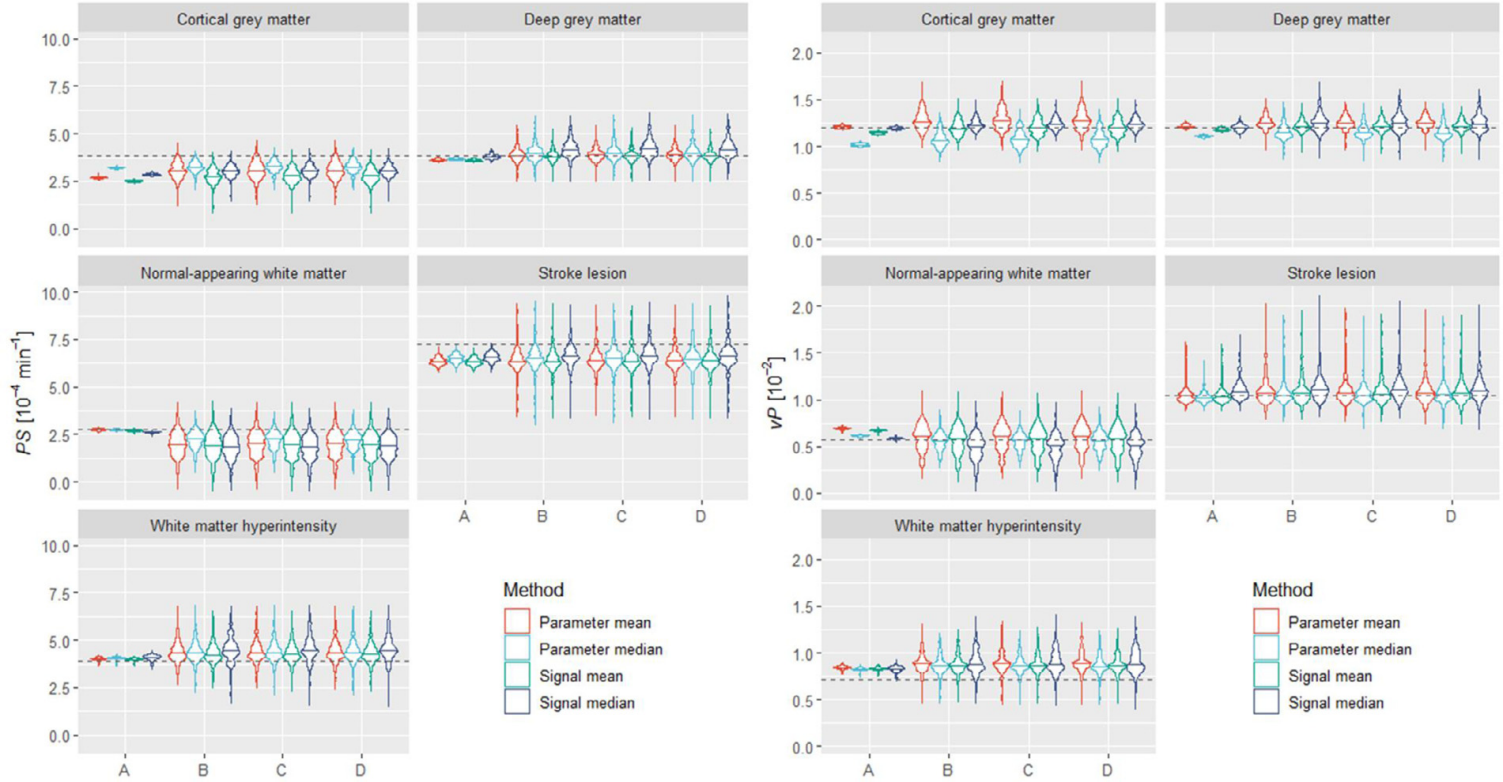
*PS* and *v*_P_ values estimated per tissue as we progressively induced spatio-temporal effects (A: *k*-space sampling only. B: sampling and gross motion. C: sampling, gross motion, and motion artefacts. D: sampling, gross motion, motion artefacts and noise). We produced these results using spatially realigned images (and without erosion or filtering). The maximum width of the violin plots was kept constant. The dotted lines represent the true *PS* and *v*_P_ values for each tissue.

To predict the accuracy and precision of quantitative *PS* and *v_P_* measurements, we performed 201 simulations of the same individual, using a different motion trajectory, noise contribution and initial orientation each time. For each run, tissue parameter values were estimated using both a signal-averaging (i.e. analysis of the tissue-averaged signal) and parameter-averaging (i.e. averaging over the *PS* and *v_P_* parametric maps) approach. The results are shown in Fig. 8 and Table S1. Overall, the parameter median approach resulted in comparable or slightly better estimates compared with other approaches. These methods clearly overestimated *PS* and *v_P_* for WMH and underestimated *PS* for cortical grey matter, NAWM and stroke lesion. Note these results are consistent with the single scan analyses shown in Fig. 5. Gross motion caused most of the quantification error, typically increasing both the systematic bias and the spread of values. Although incorporating both motion artefacts and noise increased the dispersion, the median value computed was not substantially affected.

#### 3.3.1 Effect of spatial realignment

**Fig. 9:**
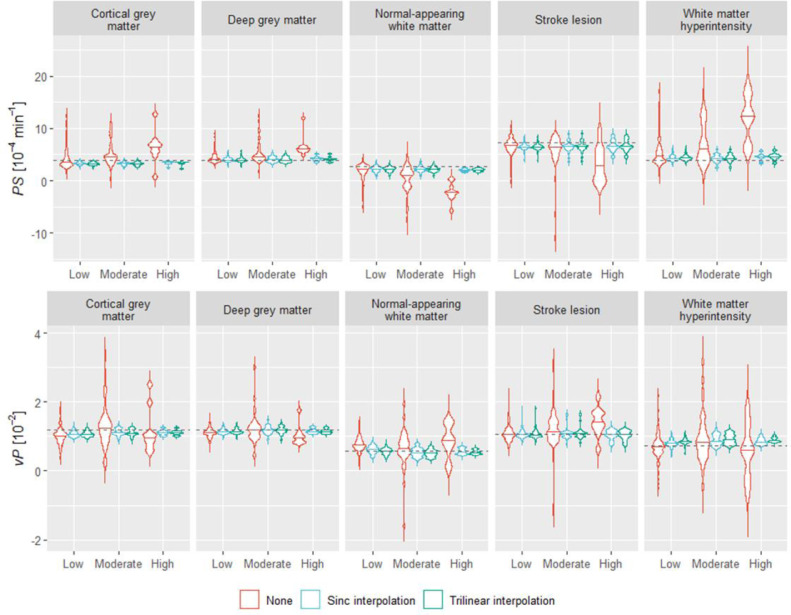
Effect of spatial realignment on parameter estimation per tissue depending on the extent of motion and interpolation method used for realignment. Estimates were obtained using the parameter median approach (without erosion or filtering). The maximum width of the violin plots was kept constant. The definition of low, moderate, and high motion can be found in the Supplementary Material. These results correspond to simulations including all spatiotemporal effects. The dotted lines represent the true *PS* and *v*_P_ values for each tissue.

Rigid-body spatial realignment is often employed to correct for head motion that may have occurred during a lengthy DCE-MRI scan (Thrippleton et al., 2019). However, not all studies in the field use or report using this pre-processing step. We compared simulation results obtained with and without spatial realignment (Fig. 9 and Table S2). These indicate that spatial realignment has a large beneficial impact on both precision and accuracy of parameter estimation, especially in cases of moderate and high motion. We additionally considered the effect of two interpolation methods: trilinear (default in MCFLIRT) and sinc interpolation. Both methods led to comparable estimation errors.

#### 3.3.2 Effect of mask erosion and low-pass filtering

**Fig. 10:**
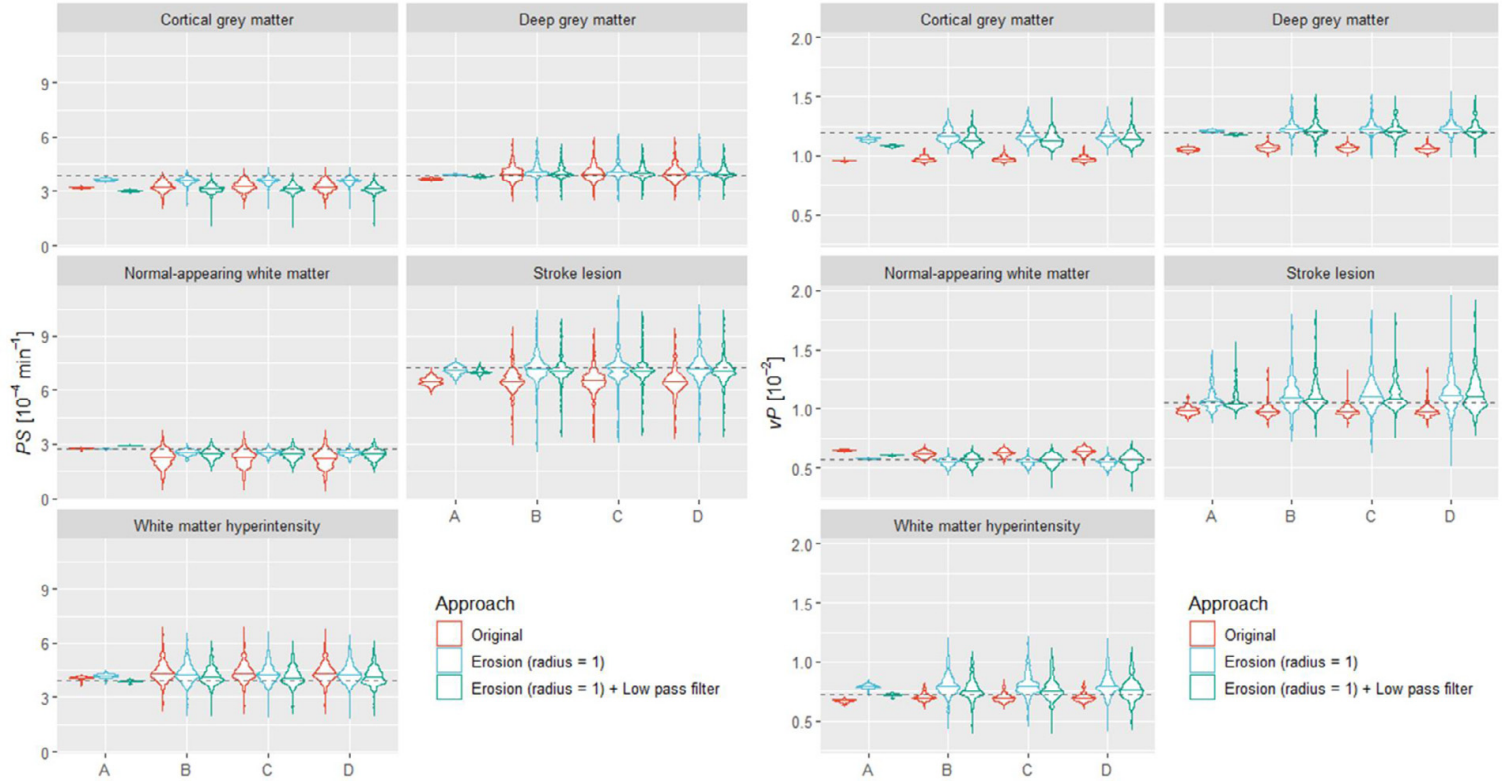
Effect of segmentation mask erosion and low pass filtering on parameter estimation per region of interest as progressively induced spatio-temporal effects (A: *k*-space sampling only. B: sampling and gross motion. C: sampling, gross motion, and motion artefacts. D: sampling, gross motion, motion artefacts and noise). Estimates were obtained following spatial realignment of the images and using the parameter map median approach. The width of the violin plots was kept constant. The dotted lines represent the true *PS* and *v*_P_ values for each tissue.

Since the results described above indicate a significant error contribution from partial volume effects and non-local signal due to Gibbs ringing, we tested whether the error decreased following erosion of the segmentation masks (Heye et al., 2016) using a sphere kernel with radius equal to one voxel. The corresponding *PS* and *v_P_* estimates showed (Fig. 10 and Table S3) increased accuracy following mask erosion in most tissues of interest. For example, when we induced all spatiotemporal effects, the relative *PS* error for NAWM and cortical grey matter was −18.47 (IQR −32.35, −10.01)% and −15.60 (IQR −19.92, −10.75)% originally versus −8.19 (−11.41, −4.76)% and −6.58 (IQR −9.18, −4.31)% following erosion.

We also tested the impact of a Bessel low pass filter (applied to the acquired *k*-space images) on parameter mapping (Figure S4) and estimation (Fig. 10 and Table S3). Simulated parameter maps demonstrate that low-pass filtering effectively mitigates ringing artefacts, while the impact on parameter estimates is similar to that of erosion.

## 4 Discussion

In this work, we present an open-source computational model for mimicking the DCE-MRI acquisition process for quantitative mapping of subtle BBB leakage under realistic conditions, including patient motion, anatomy and signal enhancement dynamics based on in-vivo clinical data. We found that even low levels of motion and image artefact have a large impact on both the appearance of parametric leakage maps and on quantitative measurements of leakage rates within regions of interest.

Previous simulation work has examined a number of other factors that may affect such measurements. For example, more than one research group has demonstrated the suitability of the Patlak pharmacokinetic model for measuring low-level *PS* (Barnes et al., 2016; Cramer and Larsson, 2014; Heye et al., 2016). The researchers also demonstrated the effects of noise, impaired cerebral blood flow, the number of pre-contrast baseline volumes acquired and the impact of scanner drift on the accuracy and precision of *PS* measurements. While such findings have provided essential insights and informed guidance regarding optimal acquisition and processing strategies (Thrippleton et al., 2019), they are based on simulations of one-dimensional time-signal data and, thus, do not address spatio-temporal factors, which, as we have shown here, have a substantial influence, over and above that of noise.

In summary, we scrutinised the impact of spatio-temporal effects on the appearance of leakage maps by progressively inducing them in the DRO. The first of these, *k*-space sampling, has two main consequences: partial volume averaging and Gibbs ringing artefact. While the appearance of Gibbs artefact on the source T1w images was subtle, the effect was greatly magnified in the leakage maps. This result can be understood by considering that, although the magnitude of ringing artefacts in the source images is merely a few percent of the signal intensity, such a signal contribution is similar to or larger than the signal changes caused by slow contrast agent leakage in brain tissue. The effect is particularly apparent in the partition direction, where the voxel dimension is greatest. Despite the Gibbs artefact, some tissue permeability differences remained apparent in the leakage maps. Inclusion of gross patient motion in the simulations further degraded the leakage maps, obscuring tissue differences and introducing artefactual features, particularly around vessels, lateral ventricles and tissue boundaries. The inclusion of motion artefact had a further impact on the quality of leakage maps, primarily in the form of ghosting and blurring features. While the incorporation of noise further degraded the appearance, the additional relative impact was small. The oval shape of some of these artefacts implies that they originate from or close to the surface of the brain, while the observed dependence on initial head position is consistent with the dependence of Gibbs artefact on the position of tissue boundaries in relation to the voxel grid (Ferreira et al., 2009). Additional simulations, in which we switched off the much larger contrast enhancement occurring in some extra-cerebral regions, suggest that finite *k*-space sampling of the high spatial frequencies induced by these enhancements has a notable impact on measured leakage patterns within the brain and is a source of the Gibbs-like artefacts seen in the parametric maps.

We also assessed the impact of the same spatio-temporal imaging factors on quantitative tissue-averaged leakage measurements. We found reasonable levels of accuracy in the absence of motion effects, with the exception of cortical grey matter and stroke lesion tissues, where sampling factors have a proportionately larger effect due to their size and morphology. Gross motion combined with *k*-space sampling had the largest effect on the accuracy and precision, consistent with the observed impact on individual leakage maps. For example, we observed a bias towards lower (−15.60 [IQR −19.92, −10.75]% and −18.47 [IQR −32.35, −10.01]%) values for cortical grey matter and normal-appearing white matter due to gross motion. Both Gibbs ringing and partial volume effects cause the mixing of signals between neighbouring tissues, which have different pre-contrast and dynamic signal intensities; in the case of motion, the degree of mixing is time-dependant, which may increase the impact on *PS* estimates, particularly if data are not spatially re-aligned. For example, the predicted underestimation of *PS* in white matter could be rationalised by the progressive “incursion” of grey-matter signal into the white matter tissue mask due to patient motion, since the size of the effect increases with degree of motion, and reduces following spatial realignment and mask erosion. Simulation of motion artefact and image noise was shown to have a much smaller effect.

Our work has implications for the interpretation of DCE-MRI subtle leakage data. First, the use of an appropriate pharmacokinetic model, temporal resolution and sufficiently high signal-to-noise ratio are essential but not sufficient conditions to obtain reliable results, since spatio-temporal considerations have a major impact on the accuracy and precision. Secondly, leakage maps in individual patients may be an unreliable source of information, as they are highly distorted by imaging artefacts and motion; such maps should be interpreted with caution, since artefactual leakage features are likely to be present, even (as simulated here) if the true leakage rate is uniform within each tissue. Therefore, it is important to acknowledge that apparent features such as leakage “hotspots”, “rims”, negative leakage rates and shifts in the distribution can be caused by patient motion and data sampling effects. This consideration is particularly relevant to studies of ageing where key pathological features, such as periventricular WMH, may coincide with regions where the artefact level is high. Post-processing techniques designed to denoise leakage maps or identify voxels with significant leakage (Raja et al., 2018; Taheri et al., 2011; van de Haar et al., 2017) could also be potentially confounded by these effects, which distort the central value, width and shape of the measured voxel leakage distribution. Third, quantitative leakage rates estimated for each tissue may also be subject to substantial systematic biases and random error as a result of these effects. Since the degree of patient motion is likely to be related to severity of neurodegenerative disease, there is therefore a possibility of inferring false associations between leakage rate and disease.

Our work also has implications for the design of future studies targeting subtle BBB leakage. The computation framework for generating DROs presented here, which is freely available as source code, provides a convenient means to evaluate and compare proposed DCE-MRI protocols including the influence of spatial and temporal resolution parameters. The critical impact of motion revealed by our experiments also argues for the following recommendations. First, the use of spatial registration is essential as its omission can lead to greatly increased systematic and random estimation errors, even in cases of low motion. Second, measures to reduce motion in the first place, such as padding of the head will be beneficial, as previously recommended (Thrippleton et al., 2019). Third, even though we obtained comparable estimates across levels of motion after spatial realignment, abrupt movements during scanning may result in image distortion, which may affect parameter mapping and estimation. Thus, we recommend checking realignment was successful in each case. Fourth, the appearance of parametric maps is improved through application of a low-pass spatial filter, albeit at the cost of image blurring. Fifth, the accuracy of quantitative parameter estimates can be improved by eroding tissue masks or regions of interest, thereby reducing signal contamination from neighbouring signals due to partial volume, Gibbs ringing and gross motion effects. Sixth, we observed similar accuracy and precision regardless of whether averaging was performed over the parameter maps or over the MR images. Nonetheless, when using parameter averaging, we recommend parameter median as it showed slightly improved estimation compared with parameter mean and it is more robust against outliers.

Future studies should evaluate the effect of motion-resistant acquisition techniques, such as optical prospective motion compensation, provided temporal resolution, signal stability and other key technical elements are not compromised. Equally, the value of post-processing techniques, such as retrospective spatial artefact reduction methods targeting truncation (Kellner et al., 2016) and motion artefacts can now also be explored.

Our work has some limitations. First, although the DROs are generated using a high-resolution (0.5-mm isotropic) brain atlas to synthesise the MRI signal, it may not be sufficient to model all anatomical structures and tissue boundaries accurately. To our knowledge, the MIDA atlas is the highest resolution head and neck atlas with comprehensive labelling available at present, however higher resolution (e.g. based on 7-T MRI) atlases could be used in future. Second, we simulated data using a number of necessary assumptions and simplifications. For example, dynamic brain tissue signals were generated using the Patlak model, since this ensures that any errors we identified are a consequence of the spatio-temporal factors investigated here and not due to physiological limitations of the model, which have been investigated extensively elsewhere. Third, our technique for simulating motion effects resulted in motion artefacts with realistic appearance within an acceptable computation time; simulating continuous patient motion (and signal enhancement changes) throughout the acquisition would generate more realistic data but at the cost of increased computation time. Fourth, the results presented herein correspond to a specific DCE-MRI protocol and would likely be quantitatively different for a different protocol. Nevertheless, the protocol simulated is typical of those described in the literature, concerning spatial resolution, acquisition time and pulse sequence. It would be straightforward to simulate alternative protocols and pulse sequences (e.g. saturation recovery spoiled gradient echo) using our framework. Fifth, we simulated a DRO including small vessel disease features seen in the ageing brain, such as WMH and stroke lesions. However, study populations typically comprise a range of disease burdens and other features, such as lacunes, enlarged perivascular spaces, micro-haemorrhages (i.e., micro-bleeds), sulcal widening and ventricular enlargement amongst others; future work could address the impact of brain ageing and neurovascular health on the accuracy of leakage measurements.

In conclusion, we have developed and made publicly-available a novel DRO for simulating DCE-MRI measurement of subtle BBB leakage (https://doi.org/10.7488/ds/2966). This development is timely, given the rapidly growing interest in neurodegenerative diseases, such as small vessel disease and dementia, which are linked with subtle BBB dysfunction, and the growing interest in applying DCE-MRI in this area. Our work reveals reasons to be careful when interpreting such data, and provides a means to estimate and optimise the reliability of measurements.

## Declaration of Competing Interest

The authors declare no conflict of interest. The funders had no role in the design of the study; in the collection, analyses, or interpretation of data; in the writing of the manuscript, or in the decision to publish the results.

## Appendix Supplementary materials

Image, application 1

## Footnotes

1 The MIDA human head model can be downloaded from www.itis.ethz.ch/MIDA/
