# Supplementary material for: A four-dimensional computational model of dynamic contrast-enhanced magnetic resonance imaging measurement of subtle blood-brain barrier leakage
Source: Neuroimage. 2021 Apr 15;230:117786. doi: 10.1016/j.neuroimage.2021.117786 (PMC8065875; doi:10.1016/j.neuroimage.2021.117786)
Supplement: Supplementary file 1 [file mmc1.docx]

A four-dimensional computational model of dynamic contrast-enhanced magnetic resonance imaging measurement of subtle blood-brain barrier leakage: supplementary material

Jose Bernal^1^, Maria d. C. Valdés-Hernández^1^, Javier Escudero^2^, Anna K. Heye^1^, Eleni Sakka^1^, Paul A. Armitage^3^, Stephen Makin^4^, Rhian M. Touyz^5^, Joanna M. Wardlaw^1^, Michael J. Thrippleton^1^

^1^Centre for Clinical Brain Sciences, Dementia Research Institute at the University of Edinburgh, Edinburgh EH16 4SB, UK

^2^School of Engineering, University of Edinburgh, Edinburgh EH9 3FB, UK

^3^Academic Unit of Radiology, University of Sheffield, Sheffield S10 2RX, UK

^4^University of Aberdeen, Centre for Rural Health, Inverness, UK

^5^Institute of Cardiovascular and Medical Sciences, University of Glasgow, Glasgow G12 8TA, UK

# A Data preparation

The MIDA model is not included as part of our package due to its License Agreement. However, the following steps should allow the user to create the segmentation map used in the present work:

- Download our computational model from https://doi.org/10.7488/ds/2966.
- Download SPM12 from https://www.fil.ion.ucl.ac.uk/spm/software/spm12 and extract it inside the “Software” folder.
- Download the MIDA model from itis.swiss/virtual-population/regional-human-models/mida-model/ and extract all files inside “input” folder of the DCEDRO package. The MIDA model should be in the following relative location: *input/MIDA_v1.0/MIDA_v1_voxels/MIDA_v1.nii.*
- Run Step_00_createSegMap.m to generate the version of the MIDA model that we used in our simulations. The resulting segmentation maps should be created in the folder “input” and should contain 17 regions of interest.

# B Distribution of healthy and pathological regions considered in our computational model

We developed the signal model based on a three-dimensional high-resolution (0.5-mm isotropic), comprehensively-labelled and publicly-available human head and neck atlas (Iacono et al., 2015)^[[1]](#footnote-1)^, and included white matter hyperintensities and lacunar stroke lesions to better represent the ageing brain. The spatial distribution of these regions in central axial slices is displayed in Figure S1.


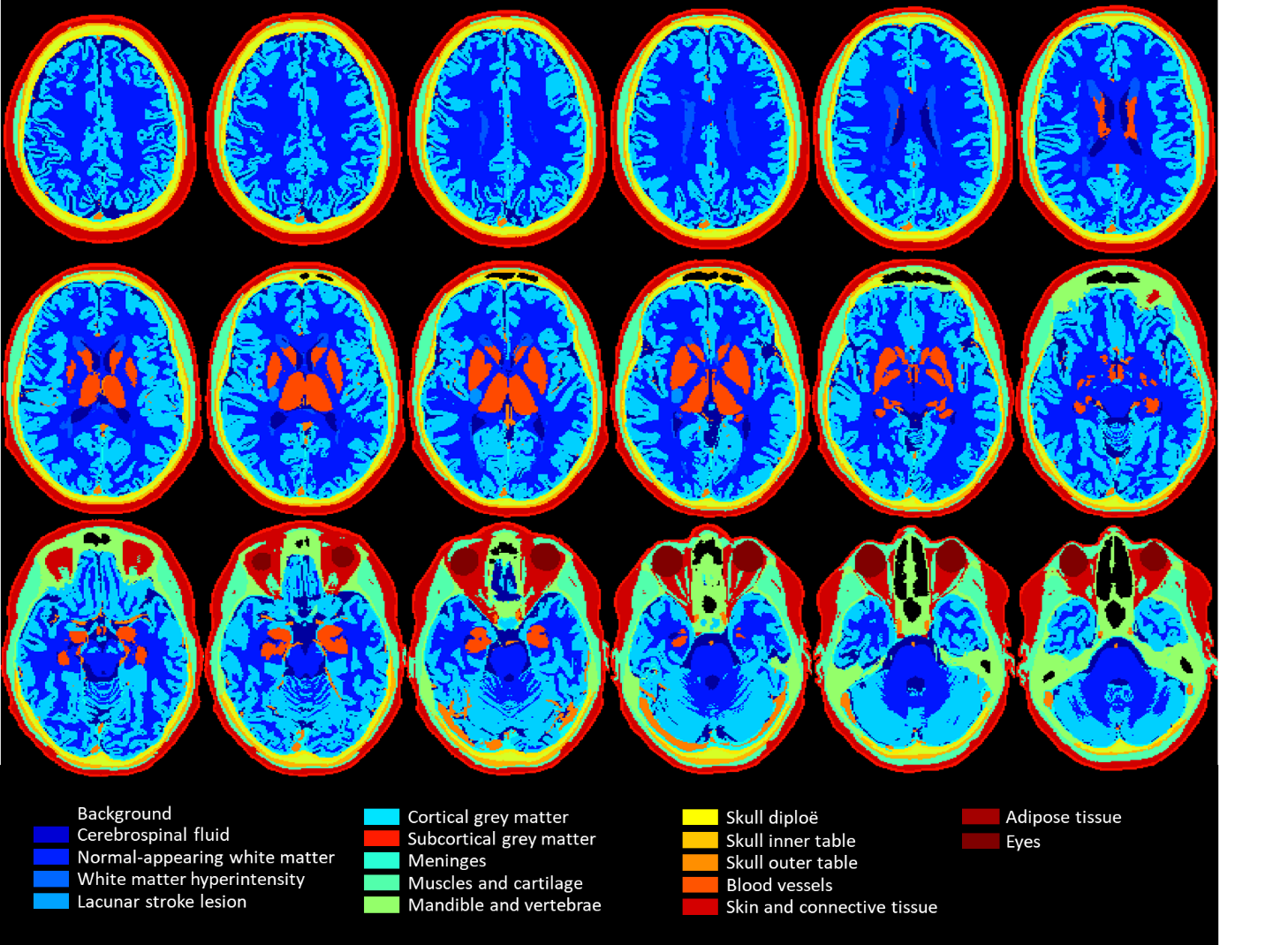


Figure S1. Central slices of the segmentation map used in our computational model showing the distribution of healthy and pathological cerebral and extra-cerebral tissues. White matter hyperintensities are located mostly periventricularly and the lacunar stroke lesion in the basal ganglia.

# C Classification of motion trajectories

We defined low, medium, and high degrees of motion based on the root mean square (RMS) voxel displacement for available trajectories in the Mild Stroke Study II. The process was two-fold. First, we computed the RMS difference between consecutive time points using the rmsdiff utility function in the FSL package and averaged the results over all time steps. In our sample, the mean RMS was approximately 0.33 (IQR 0.22 – 0.48) mm for each inter-frame step. Second, we classified all 201 trajectories according to the following criteria:

- Low motion (n=150): mean RMS lower than 0.48 mm [< Q3];
- Moderate motion (n=41): mean RMS between 0.48 mm and 0.84 mm [Q3 < mean RMS < Q3 + 1.5*IQR];
- High motion (n=10): mean RMS higher than 0.84 mm [> Q3 + 1.5*IQR].

# D Signal-time curves of cerebral and extra-cerebral regions

We generated signal-time curves based on in-vivo patient data (MSS2 study). For brain tissues, we used measured pharmacokinetic parameters (Heye et al., 2016) and the Patlak model to generate their signal-time curves. For simulating enhancement in extracerebral regions, we generated synthetic signal-time curves based on direct signal measurements from in-vivo patient data. First, we sampled signal-time curves for meninges, muscles and cartilage, mandible and vertebrae, skull diploe, skull inner table, skull inner table, skin and connective tissue, adipose tissue, and eyes manually, following recommendations from an experienced neuroradiologist. Second, we fitted exponential or power functions to the extracted signal-time curves to remove noise. The fitted functions can be found in Utils/getNonBrainSignals.m.

The input signal-time curves for both cerebral and extra-cerebral tissues can be seen in Figure S2. All brain tissues enhance over time after contrast agent administration. In our simulations, the maximum enhancement for normal-appearing white matter, white matter hyperintensity, stroke lesion and grey matter was approximately 7%, 12%, 18% and 22%, respectively. Except for skull diploe, adipose tissue and eyes, the signal in extra-cerebral regions enhanced by up to 60%.


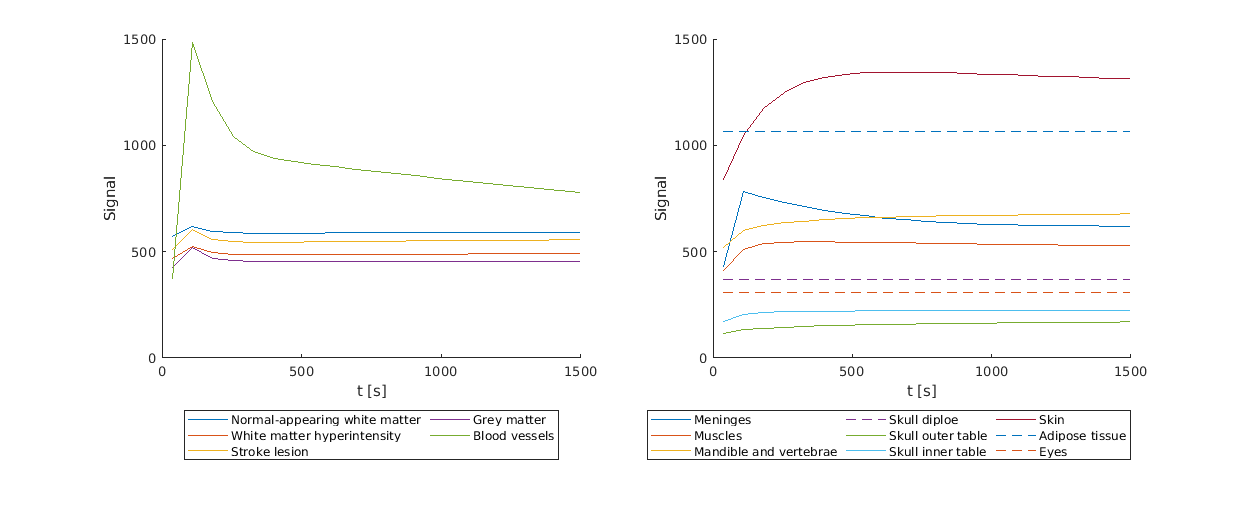


Figure S2. Ground-truth signal-time curves generated in cerebral and extra-cerebral tissues based on in-vivo data.

# E Effect of slice thickness on parameter maps

To evaluate the effect of slice thickness on the appearance of parameter maps, we computed the *PS* and *v_P_* maps with slice thickness equal to 2 mm and 4 mm on a single DRO (Figure S3). Qualitatively, the use of thinner slices results in reduced ringing artefacts. Quantitatively, relative error in the parameters is typically reduced at 2-mm vs. 4-mm slice thickness. For the simulated case and in the absence of motion and noise, the effect was particularly strong in cortical grey matter, where the relative error reduces from -16.72% and -13.97% when using 4 mm slices to -0.06 % and -1.04 % when using 2 mm slices, for *PS* and *v*_P_ respectively.


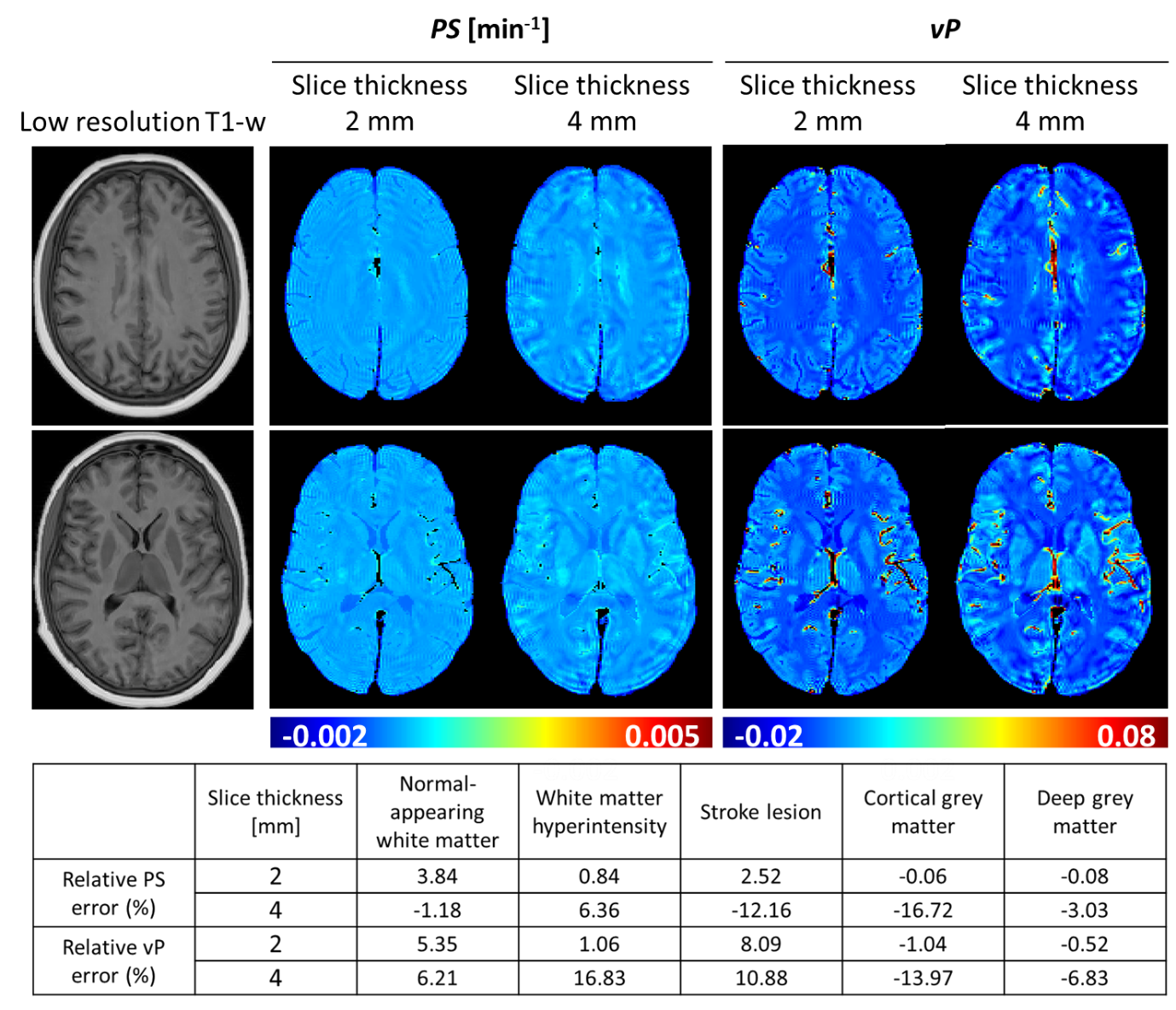


Figure S3. Effect of slice thickness on the appearance of PS and v_P_ maps and their quantification. We considered a single DRO and two thicknesses: two and four millimetres. Thinner slices result in reduced ringing artefacts and lower parameter estimation error overall. The numbers correspond to median voxel value per region of interest. Simulations excluded motion and noise effects.

# F Effect of low-pass *k*-space filtering on parameter maps

Gibbs ringing artefacts can be diminished by applying a low-pass filter to the acquired *k*-space. We implemented a Bessel low-pass filter (filter order 5; cut-off frequency: half low-resolution field of view in *k*-space), and examined its effect on parameter mapping. The application of a low-pass filter enhances the qualitative appearance of both *PS* and *v_P_* maps, reducing the appearance of ringing artefacts (Figure S4).


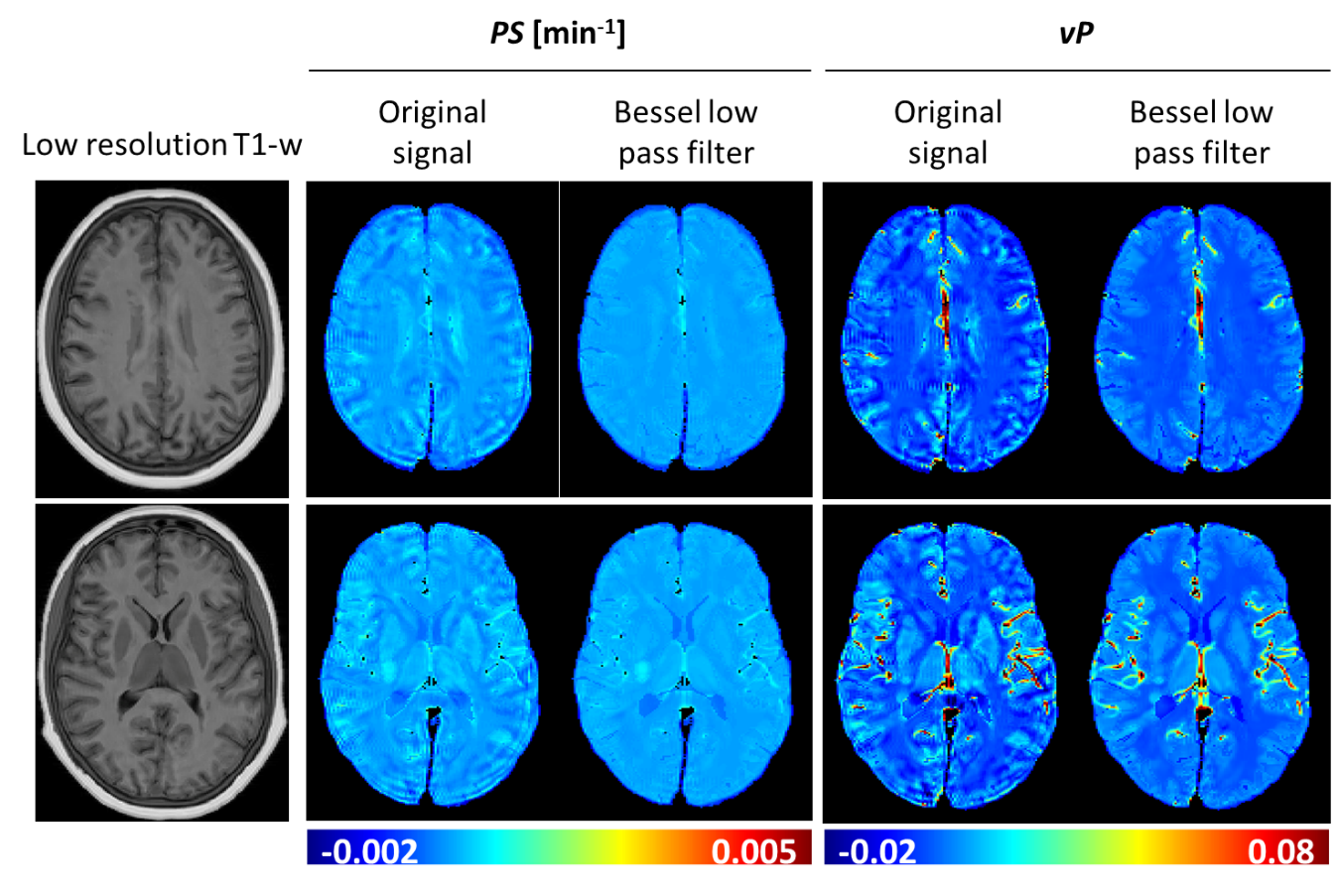


Figure S4. Effect of applying Bessel low pass filter to acquired k-space data on the appearance of PS and v_P_ maps. The low pass filter can help to mitigate ringing artefacts, as it attenuates high spatial frequencies. Simulations included gross motion but excluded motion artefacts and noise effects. The extent of motion was low.

# G Accuracy and precision of quantitative parameter estimation

Table S1. Relative parameter estimation error obtained using parameter map and signal averaging approaches as we progressively induced spatiotemporal effects. Here we report median error and interquartile range for the simulations results presented in Figure 8. NAWM: normal-appearing white matter. WMH: white matter hyperintensity. SL: stroke lesion. CGM: cortical grey matter. DGM: deep grey matter.

| Spatiotemporal  consideration | Method | Tissue | Relative *PS* error (%) | Relative v_P_ error (%) |
| --- | --- | --- | --- | --- |
| Sampling | Parameter mean | CGM | -30.39 (-31.55, -29.79) | 0.93 (-0.05, 1.68) |
|  |  | DGM | -5.97 (-6.82, -4.82) | 1.08 (0.37, 2.64) |
|  |  | NAWM | 0.08 (-1.44, 1.1) | 21.71 (20.84, 22.53) |
|  |  | SL | -12.67 (-14.09, -10.73) | -0.81 (-4.58, 3.63) |
|  |  | WMH | 2.96 (2.3, 4.27) | 17.31 (15.99, 18.71) |
|  | Parameter median | CGM | -17.05 (-17.75, -16.55) | -15.11 (-16.08, -14.21) |
|  |  | DGM | -4.56 (-5.26, -3.53) | -7.36 (-7.94, -6.95) |
|  |  | NAWM | 0.45 (-0.39, 1.11) | 7.91 (6.93, 8.66) |
|  |  | SL | -10.32 (-12.38, -8.42) | -3.07 (-5.3, 0.11) |
|  |  | WMH | 4.41 (3.69, 5.16) | 14.88 (14.02, 16) |
|  | Signal mean | CGM | -35 (-36.15, -34.29) | -4.18 (-5.16, -3.49) |
|  |  | DGM | -6.58 (-7.41, -5.6) | -1.55 (-2.22, -0.28) |
|  |  | NAWM | -1.1 (-2.68, -0.03) | 17.94 (17.07, 18.68) |
|  |  | SL | -13.02 (-14.38, -10.99) | -1.25 (-4.81, 3.16) |
|  |  | WMH | 2.14 (1.57, 3.21) | 15.5 (14.32, 16.68) |
|  | Signal median | CGM | -25.93 (-26.63, -25) | -0.47 (-1.02, 0.38) |
|  |  | DGM | -1.44 (-3.35, 0.14) | 0.34 (-1.84, 1.94) |
|  |  | NAWM | -4.99 (-5.79, -4.45) | 2.69 (1.94, 3.6) |
|  |  | SL | -9.33 (-11.35, -7.06) | 3.63 (-1.08, 9.13) |
|  |  | WMH | 5.06 (2.93, 6.77) | 15.33 (12.82, 17.79) |
| Sampling and gross motion | Parameter mean | CGM | -21.18 (-27.87, -12.68) | 5.71 (1.13, 11.73) |
|  |  | DGM | 0.49 (-3.85, 5.35) | 3.83 (1.33, 7.84) |
|  |  | NAWM | -28.46 (-44.14, -10.75) | 7.41 (-12.24, 21.02) |
|  |  | SL | -12.29 (-15.34, -7.74) | 2.06 (-3.25, 7.86) |
|  |  | WMH | 10.3 (3.45, 18.74) | 22.91 (16.16, 30.08) |
|  | Parameter median | CGM | -15.48 (-19.02, -10.77) | -11.34 (-14.64, -6.33) |
|  |  | DGM | 2.81 (-2.91, 9.33) | -4.34 (-7.38, 0.58) |
|  |  | NAWM | -17.4 (-29.63, -8.07) | -0.51 (-13.47, 7.23) |
|  |  | SL | -10.22 (-13.93, -5.77) | 0.1 (-4.14, 4.58) |
|  |  | WMH | 10.38 (4.29, 18.95) | 19.38 (13.39, 26.26) |
|  | Signal mean | CGM | -28.88 (-34.01, -21.61) | -0.78 (-4.73, 4.2) |
|  |  | DGM | -1.23 (-5.06, 2.95) | 1.18 (-1.17, 4.29) |
|  |  | NAWM | -30.04 (-46.53, -12.34) | 3.4 (-17.53, 17.33) |
|  |  | SL | -12.63 (-15.67, -8.12) | 1.59 (-3.52, 7.22) |
|  |  | WMH | 8.85 (2.23, 16.43) | 20.57 (14.32, 27.55) |
|  | Signal median | CGM | -21.37 (-25.66, -16.61) | 2.49 (0.1, 6.6) |
|  |  | DGM | 8.39 (1.54, 15.63) | 4.17 (-0.66, 9.01) |
|  |  | NAWM | -32.34 (-47.64, -17.19) | -11.45 (-29.4, 1.41) |
|  |  | SL | -8.76 (-12.03, -3.74) | 5.74 (-0.34, 12.88) |
|  |  | WMH | 14.51 (6.38, 23.6) | 21.88 (14.46, 33.68) |
| Sampling, gross motion and motion artefacts | Parameter mean | CGM | -20.08 (-27.63, -11.83) | 6.16 (1.12, 13.14) |
|  |  | DGM | 0.62 (-3.41, 5.34) | 4.41 (1.34, 7.89) |
|  |  | NAWM | -27.08 (-44.03, -11.95) | 8.01 (-12.88, 20.7) |
|  |  | SL | -12.12 (-15.38, -7.53) | 1.74 (-3.2, 8.19) |
|  |  | WMH | 10.16 (4.65, 19.57) | 22.81 (16.92, 31.4) |
|  | Parameter median | CGM | -15.04 (-19.23, -10.45) | -10.6 (-14.97, -5.11) |
|  |  | DGM | 2.71 (-2.07, 8.9) | -4.61 (-7.2, 0.05) |
|  |  | NAWM | -16.78 (-30.16, -9.2) | 0.24 (-13.11, 7.46) |
|  |  | SL | -9.9 (-13.89, -5.79) | 0.14 (-4.63, 4.85) |
|  |  | WMH | 11 (4.77, 19.2) | 19.58 (14.74, 28.31) |
|  | Signal mean | CGM | -27.57 (-33.63, -21.45) | -0.34 (-4.43, 4.84) |
|  |  | DGM | -0.88 (-4.44, 3.09) | 1.35 (-1.19, 4.4) |
|  |  | NAWM | -29.08 (-46.88, -13.97) | 4.25 (-17.56, 16.74) |
|  |  | SL | -12.41 (-15.58, -7.97) | 1.25 (-3.57, 7.3) |
|  |  | WMH | 9.42 (2.93, 17.32) | 20.37 (15.07, 28.33) |
|  | Signal median | CGM | -21.18 (-25.73, -15.99) | 2.92 (0.18, 6.79) |
|  |  | DGM | 9.09 (1.82, 16.85) | 3.65 (-0.54, 8.59) |
|  |  | NAWM | -34.26 (-50.45, -17.23) | -10.04 (-30.13, 0.35) |
|  |  | SL | -8.48 (-12.13, -3.38) | 5.14 (-0.36, 12.29) |
|  |  | WMH | 14.17 (7.29, 25.17) | 21.34 (14.51, 36.64) |
| Sampling, gross motion, motion artefacts and noise | Parameter mean | CGM | -19.96 (-27.33, -11.78) | 6.4 (1.36, 13.24) |
|  |  | DGM | 0.71 (-3.17, 5.45) | 4.55 (1.39, 7.85) |
|  |  | NAWM | -26.63 (-43.93, -11.73) | 8.06 (-12.32, 20.95) |
|  |  | SL | -11.82 (-15.27, -7.22) | 2.02 (-2.86, 9.1) |
|  |  | WMH | 11.02 (4.93, 19.55) | 23.16 (17.75, 32.34) |
|  | Parameter median | CGM | -15.6 (-19.92, -10.75) | -10.39 (-14.91, -4.76) |
|  |  | DGM | 2.5 (-2.46, 8.6) | -4.93 (-7.88, -0.23) |
|  |  | NAWM | -18.47 (-32.35, -10.01) | 0.26 (-14.91, 9.05) |
|  |  | SL | -10.91 (-14.59, -6.62) | 0.65 (-4.55, 5.96) |
|  |  | WMH | 10.37 (3.95, 19.03) | 19.01 (14.62, 27.67) |
|  | Signal mean | CGM | -27.76 (-33.47, -21.41) | -0.24 (-4.29, 4.88) |
|  |  | DGM | -0.92 (-4.26, 3.04) | 1.42 (-1.26, 4.34) |
|  |  | NAWM | -28.91 (-46.78, -14.11) | 4.04 (-17.63, 16.59) |
|  |  | SL | -12.21 (-15.71, -7.64) | 1.47 (-3.38, 8.16) |
|  |  | WMH | 9.23 (3.26, 16.97) | 20.36 (15.55, 28.86) |
|  | Signal median | CGM | -21.04 (-25.62, -16.19) | 3.03 (0.23, 6.67) |
|  |  | DGM | 7.68 (1.61, 15.68) | 3.29 (-0.97, 8.47) |
|  |  | NAWM | -31.83 (-48, -15.97) | -9.43 (-27.93, 1.23) |
|  |  | SL | -8.34 (-12.71, -3.84) | 4.26 (-1.12, 12.19) |
|  |  | WMH | 14.43 (6.24, 25.34) | 21.43 (13.75, 36.73) |

Table S2. Relative parameter estimation error obtained using parameter map median depending on the extent of motion. Here we report median error and interquartile range for the simulations results presented in Figure 9. NAWM: normal-appearing white matter. WMH: white matter hyperintensity. SL: stroke lesion. CGM: cortical grey matter. DGM: deep grey matter.

| Motion | Similarity metric | Tissue | Relative *PS* error (%) | Relative v_P_ error (%) |
| --- | --- | --- | --- | --- |
| Low | None | CGM | -9.01 (-21.9, 27.15) | -14.22 (-27.07, -6.83) |
|  |  | DGM | 5.95 (-2.17, 21.38) | -8.07 (-14.23, -2.95) |
|  |  | NAWM | -19.73 (-50.22, -0.05) | 30.42 (14.64, 56.73) |
|  |  | SL | -7.67 (-19.13, 2.44) | 2.03 (-9.09, 15.1) |
|  |  | WMH | 20.68 (-4.22, 55.98) | -2.68 (-24.19, 17.87) |
|  | Sinc interpolation | CGM | -14.74 (-17.5, -8.88) | -12.25 (-16.01, -6.69) |
|  |  | DGM | 3.01 (-1.43, 10.33) | -5.66 (-8.72, -1.44) |
|  |  | NAWM | -16.96 (-32.21, -7.2) | 8.72 (-4.55, 20.94) |
|  |  | SL | -11.04 (-13.83, -7.35) | 0.44 (-4.7, 5.77) |
|  |  | WMH | 6.08 (0.71, 18.29) | 10.82 (3.73, 17.83) |
|  | Trilinear interpolation | CGM | -15.92 (-19.95, -10.98) | -11.8 (-15.47, -6.48) |
|  |  | DGM | 1.92 (-2.26, 7.85) | -5.48 (-8.05, -1.08) |
|  |  | NAWM | -18.29 (-32.34, -9.73) | 1.13 (-12.26, 10.73) |
|  |  | SL | -11.14 (-14.01, -7.11) | -0.14 (-4.63, 5.52) |
|  |  | WMH | 10.38 (4.29, 17.37) | 17.88 (14.61, 23.87) |
| Moderate | None | CGM | 18.49 (0.66, 49.64) | 5.76 (-23.07, 23.13) |
|  |  | DGM | 19.32 (8.19, 44.7) | -2.12 (-16.77, 10.03) |
|  |  | NAWM | -59.04 (-136.23, -28.41) | 11.16 (-19.88, 60.52) |
|  |  | SL | -11.71 (-31.81, 4.82) | 6.84 (-12.06, 36.82) |
|  |  | WMH | 49.09 (3.91, 165.5) | 15.72 (-34.16, 72.72) |
|  | Sinc interpolation | CGM | -14.59 (-20.49, -10.56) | -8.19 (-11.56, -0.1) |
|  |  | DGM | 4.73 (-4.65, 13.19) | -1.23 (-6.81, 4.16) |
|  |  | NAWM | -18.57 (-30.51, -8.49) | -6.93 (-22.91, 10.9) |
|  |  | SL | -9.55 (-15.93, -6.11) | 2.39 (-4.89, 6.56) |
|  |  | WMH | 7.57 (-0.51, 15.85) | 16.63 (5.62, 41.33) |
|  | Trilinear interpolation | CGM | -15.01 (-21.72, -11.56) | -9.4 (-12.02, -0.09) |
|  |  | DGM | 2.99 (-5.05, 13.74) | -1.54 (-6.47, 2.35) |
|  |  | NAWM | -18.55 (-31.4, -11.56) | -9.44 (-22.68, 2.37) |
|  |  | SL | -9.55 (-15.7, -6.4) | 2.21 (-4.19, 6) |
|  |  | WMH | 8.23 (3.11, 20.39) | 25.61 (10.52, 43.45) |
| High | None | CGM | 69.84 (44.27, 84.13) | -19.87 (-36.5, -10.06) |
|  |  | DGM | 57.12 (46.7, 67.96) | -19.45 (-29.71, -12.69) |
|  |  | NAWM | -181.28 (-201.45, -151.19) | 51.73 (16.8, 122.57) |
|  |  | SL | -60.38 (-94.38, -26.39) | 33.19 (18.85, 59.24) |
|  |  | WMH | 214.7 (107.65, 291.33) | -19.5 (-89.25, 45.81) |
|  | Sinc interpolation | CGM | -8.87 (-13.19, -7.07) | -7.32 (-10.77, -4.97) |
|  |  | DGM | 9.89 (3.6, 12.51) | -4.46 (-7.14, -0.98) |
|  |  | NAWM | -25.48 (-28.53, -20.79) | -2.36 (-14.29, 12.44) |
|  |  | SL | -9.99 (-15.05, -0.04) | -0.22 (-6.65, 9.58) |
|  |  | WMH | 18.83 (12.28, 21.16) | 16.49 (8.03, 26.94) |
|  | Trilinear interpolation | CGM | -10.52 (-14.12, -8.39) | -8.01 (-10.11, -4.91) |
|  |  | DGM | 7.7 (3.26, 9.66) | -5.08 (-7.39, -1.7) |
|  |  | NAWM | -23.51 (-32.38, -18.66) | -6.89 (-14.97, 1.4) |
|  |  | SL | -9.84 (-15.17, -0.1) | -0.58 (-7.88, 8.55) |
|  |  | WMH | 19.32 (10.46, 25.73) | 20.19 (18.22, 31.66) |

Table S3. Relative parameter estimation error obtained using parameter map median and post-processing strategies as we progressively induced spatiotemporal effects. Here we report median error and interquartile range for the simulations results presented in Figure 10. NAWM: normal-appearing white matter. WMH: white matter hyperintensity. SL: stroke lesion. CGM: cortical grey matter. DGM: deep grey matter.

| Spatiotemporal consideration | Post-processing | Tissue | Relative *PS* error (%) | Relative v_P_ error (%) |
| --- | --- | --- | --- | --- |
| Sampling | None | CGM | -17.05 (-17.75, -16.55) | -15.11 (-16.08, -14.21) |
|  |  | DGM | -4.56 (-5.26, -3.53) | -7.36 (-7.94, -6.95) |
|  |  | NAWM | 0.45 (-0.39, 1.11) | 7.91 (6.93, 8.66) |
|  |  | SL | -10.32 (-12.38, -8.42) | -3.07 (-5.3, 0.11) |
|  |  | WMH | 4.41 (3.69, 5.16) | 14.88 (14.02, 16) |
|  | Erosion  (radius = 1) | CGM | -5.7 (-6.68, -4.72) | -4.85 (-5.9, -4.02) |
|  |  | DGM | 1.37 (0.96, 1.83) | 0.92 (0.49, 1.31) |
|  |  | NAWM | -0.21 (-0.81, 0.31) | 1.4 (0.73, 2) |
|  |  | SL | -1.84 (-3.83, 0.44) | 0.94 (-1.4, 6.24) |
|  |  | WMH | 7.4 (5.71, 8.97) | 9.88 (8.68, 11.43) |
|  | Erosion  (radius = 1)  Low pass filter | CGM | -21.52 (-22.4, -20.82) | -9.48 (-10.01, -9.2) |
|  |  | DGM | -1.08 (-1.59, -0.19) | -1.74 (-1.93, -1.37) |
|  |  | NAWM | 6.69 (6.45, 6.87) | 7.17 (6.68, 7.39) |
|  |  | SL | -3.46 (-3.95, -1.83) | -1.1 (-2.52, 5.62) |
|  |  | WMH | -0.34 (-0.73, 0.17) | 0.5 (-0.03, 1.02) |
| Sampling and gross motion | None | CGM | -15.48 (-19.02, -10.77) | -11.34 (-14.64, -6.33) |
|  |  | DGM | 2.81 (-2.91, 9.33) | -4.34 (-7.38, 0.58) |
|  |  | NAWM | -17.4 (-29.63, -8.07) | -0.51 (-13.47, 7.23) |
|  |  | SL | -10.22 (-13.93, -5.77) | 0.1 (-4.14, 4.58) |
|  |  | WMH | 10.38 (4.29, 18.95) | 19.38 (13.39, 26.26) |
|  | Erosion  (radius = 1) | CGM | -6.46 (-8.95, -4.37) | -3.09 (-5.34, 0.29) |
|  |  | DGM | 4.75 (1.37, 10.28) | 2.32 (0.77, 4.62) |
|  |  | NAWM | -6.79 (-10.2, -3.59) | -2.94 (-6.93, -0.29) |
|  |  | SL | -0.63 (-5.03, 3.8) | 4.3 (-0.77, 11.42) |
|  |  | WMH | 8.57 (2.48, 17.9) | 9.8 (4.12, 17.41) |
|  | Erosion  (radius = 1)  Low pass filter | CGM | -18.6 (-21.54, -15.5) | -6.15 (-8.14, -2.52) |
|  |  | DGM | 2.58 (0.26, 9.24) | 0.8 (-1.08, 3.54) |
|  |  | NAWM | -9.85 (-16.76, -4.71) | -0.13 (-4.52, 4.13) |
|  |  | SL | -2.38 (-5.33, 0.17) | 2.64 (-0.22, 10.39) |
|  |  | WMH | 4.54 (-0.76, 12.85) | 4.55 (0.37, 12.55) |
| Sampling, gross motion and motion artefacts | None | CGM | -15.04 (-19.23, -10.45) | -10.6 (-14.97, -5.11) |
|  |  | DGM | 2.71 (-2.07, 8.9) | -4.61 (-7.2, 0.05) |
|  |  | NAWM | -16.78 (-30.16, -9.2) | 0.24 (-13.11, 7.46) |
|  |  | SL | -9.9 (-13.89, -5.79) | 0.14 (-4.63, 4.85) |
|  |  | WMH | 11 (4.77, 19.2) | 19.58 (14.74, 28.31) |
|  | Erosion  (radius = 1) | CGM | -6.14 (-8.88, -4.07) | -2.99 (-5.17, 0.65) |
|  |  | DGM | 4.89 (1.9, 10.31) | 2.3 (0.53, 4.59) |
|  |  | NAWM | -6.9 (-10.67, -3.74) | -2.66 (-6.98, -0.53) |
|  |  | SL | -0.37 (-5.44, 3.38) | 4.93 (-0.41, 11.78) |
|  |  | WMH | 8.52 (1.81, 17.32) | 10.58 (4.47, 16.47) |
|  | Erosion  (radius = 1)  Low pass filter | CGM | -18.58 (-21.03, -15.07) | -5.9 (-8.47, -2.39) |
|  |  | DGM | 3.27 (0.39, 9.08) | 0.75 (-1.31, 3.32) |
|  |  | NAWM | -10.36 (-17.74, -4.72) | 0.2 (-4.57, 4.83) |
|  |  | SL | -2.45 (-5.19, 0.5) | 2.7 (-0.98, 10.61) |
|  |  | WMH | 4.37 (-1.25, 12.84) | 5.44 (-0.18, 12.63) |
| Sampling, gross motion, motion artefacts and noise | None | CGM | -15.6 (-19.92, -10.75) | -10.39 (-14.91, -4.76) |
|  |  | DGM | 2.5 (-2.46, 8.6) | -4.93 (-7.88, -0.23) |
|  |  | NAWM | -18.47 (-32.35, -10.01) | 0.26 (-14.91, 9.05) |
|  |  | SL | -10.91 (-14.59, -6.62) | 0.65 (-4.55, 5.96) |
|  |  | WMH | 10.37 (3.95, 19.03) | 19.01 (14.62, 27.67) |
|  | Erosion  (radius = 1) | CGM | -6.58 (-9.18, -4.31) | -2.93 (-5.02, 0.7) |
|  |  | DGM | 4.7 (2.11, 10.07) | 2.34 (0.57, 4.74) |
|  |  | NAWM | -8.19 (-11.41, -4.76) | -3.93 (-8.15, -0.79) |
|  |  | SL | -0.73 (-4.45, 4.11) | 5.95 (0.81, 13.5) |
|  |  | WMH | 8.59 (1.47, 16.14) | 9.3 (4.3, 17.69) |
|  | Erosion  (radius = 1)  Low pass filter | CGM | -18.68 (-21.11, -15.18) | -5.42 (-7.7, -2.05) |
|  |  | DGM | 2.8 (0.34, 8.98) | 0.43 (-1.23, 3.86) |
|  |  | NAWM | -10.61 (-17.74, -5) | -0.04 (-6.26, 5.73) |
|  |  | SL | -2.47 (-6.07, 1.43) | 4.31 (-1.72, 13.67) |
|  |  | WMH | 4.7 (-1.74, 14.14) | 5.58 (-0.81, 12.53) |

# References

Heye, A.K., Thrippleton, M.J., Armitage, P.A., Valdés Hernández, M. del C., Makin, S.D., Glatz, A., Sakka, E., Wardlaw, J.M., 2016. Tracer kinetic modelling for DCE-MRI quantification of subtle blood-brain barrier permeability. Neuroimage 125, 446–455. https://doi.org/10.1016/j.neuroimage.2015.10.018

Iacono, M.I., Neufeld, E., Akinnagbe, E., Bower, K., Wolf, J., Oikonomidis, I.V., Sharma, D., Lloyd, B., Wilm, B.J., Wyss, M., Pruessmann, K.P., Jakab, A., Makris, N., Cohen, E.D., Kuster, N., Kainz, W., Angelone, L.M., 2015. MIDA: A multimodal imaging-based detailed anatomical model of the human head and neck. PLoS One 10. https://doi.org/10.1371/journal.pone.0124126

1. The MIDA human head model can be downloaded from *www.itis.ethz.ch/MIDA/* [↑](#footnote-ref-1)
